# A Comprehensive Benchmark of Kernel Methods to Extract Protein–Protein Interactions from Literature

**DOI:** 10.1371/journal.pcbi.1000837

**Published:** 2010-07-01

**Authors:** Domonkos Tikk, Philippe Thomas, Peter Palaga, Jörg Hakenberg, Ulf Leser

**Affiliations:** 1 Knowledge Management in Bioinformatics, Computer Science Department, Humboldt-Universität zu Berlin, Berlin, Germany; 2 Department of Telecommunications and Media Informatics, Budapest University of Technology and Economics, Budapest, Hungary; 3 Department of Computer Science and Engineering, Arizona State University, Tempe, Arizona, United States of America; University of Chicago, United States of America

## Abstract

The most important way of conveying new findings in biomedical research is scientific publication. Extraction of protein–protein interactions (PPIs) reported in scientific publications is one of the core topics of text mining in the life sciences. Recently, a new class of such methods has been proposed - convolution kernels that identify PPIs using deep parses of sentences. However, comparing published results of different PPI extraction methods is impossible due to the use of different evaluation corpora, different evaluation metrics, different tuning procedures, etc. In this paper, we study whether the reported performance metrics are robust across different corpora and learning settings and whether the use of deep parsing actually leads to an increase in extraction quality. Our ultimate goal is to identify the one method that performs best in real-life scenarios, where information extraction is performed on unseen text and not on specifically prepared evaluation data. We performed a comprehensive benchmarking of nine different methods for PPI extraction that use convolution kernels on rich linguistic information. Methods were evaluated on five different public corpora using cross-validation, cross-learning, and cross-corpus evaluation. Our study confirms that kernels using dependency trees generally outperform kernels based on syntax trees. However, our study also shows that only the best kernel methods can compete with a simple rule-based approach when the evaluation prevents information leakage between training and test corpora. Our results further reveal that the F-score of many approaches drops significantly if no corpus-specific parameter optimization is applied and that methods reaching a good AUC score often perform much worse in terms of F-score. We conclude that for most kernels no sensible estimation of PPI extraction performance on new text is possible, given the current heterogeneity in evaluation data. Nevertheless, our study shows that three kernels are clearly superior to the other methods.

## Author Summary

The most important way of conveying new findings in biomedical research is scientific publication. In turn, the most recent and most important findings can only be found by carefully reading the scientific literature, which becomes more and more of a problem because of the enormous number of published articles. This situation has led to the development of various computational approaches to the automatic extraction of important facts from articles, mostly concentrating on the recognition of protein names and on interactions between proteins (PPI). However, so far there is little agreement on which methods perform best for which task. Our paper reports on an extensive comparison of nine recent PPI extraction tools. We studied their performance in various settings on a set of five different text collections containing articles describing PPIs, which for the first time allows for an unbiased comparison of their respective effectiveness. Our results show that the tools' performance depends largely on the collection they are trained on and the collection they are then evaluated on, which means that extrapolating their measured performance to arbitrary text is still highly problematic. We also show that certain classes of methods for extracting PPIs are clearly superior to other classes.

## Introduction

Protein-protein interactions (PPIs) are integral to virtually all cellular processes, such as metabolism, signaling, regulation, and proliferation. Collecting data on individual interactions is crucial for understanding these processes at a systems biology level [1]. Known PPIs help to predict the function of yet uncharacterized proteins, for instance using conserved PPI networks [2] or proximity in a PPI network [3]. Networks can be generated from molecular interaction data and are useful for multiple purposes, such as identification of functional modules [4] or finding novel associations between genes and diseases [5].

Several approaches are in use to study interactions in large- or small-scale experiments. Among the techniques most often used are two-hybrid screens, mass spectrometry, and tandem affinity purification [6]. Results of high-throughput techniques (such as two-hybrid screens and mass spectrometry) usually are published in tabular form and can be imported by renowned PPI databases quickly. These techniques are prone to produce comparably large numbers of false positives [7]. Other techniques, such as co-immunoprecipitation, cross-linking, or rate-zonal centrifugation, produce more reliable results but are small-scale; these are typically used to verify interesting yet putative interactions, possibly first hypothesized during large-scale experiments [8]. Only now, authors started to submit results directly to PPI databases in a regular manner, oftentimes as a step required by publishers to ensure quality.

Taking into account the great wealth of PPI data that was published before the advent of PPI databases, it becomes clear that still much valuable data is available only in text. Turning this information into a structured form is a costly task that has to be performed by human experts [9]. Recent years have seen a steep increase in the number of techniques that aim to alleviate this task by applying computational methods, especially machine learning and statistical natural language processing [10]. Such tools are not only used to populate PPI databases, but their output is often also used directly as independent input to biological data mining (see, e.g., [11], [12]).

Several techniques for extracting protein-protein interactions from text have been proposed (cf. Related Work). Unfortunately, the reported results differ widely. While early works reported fabulous results of over 90% precision and recall [13], the recent BioCreative II.5 community challenge led to results at the opposite edge of the quality range, with the best system performing just above 30% F-measure [14]. Much of these differences can be accounted to the fact that some evaluations work on corpora that have proteins already annotated, while others include recognition and identification of proteins as a subtask [15]. However, even within the same setting, the spread of reported results remains large. Since there also is a lack of unbiased benchmarks of published systems, a potential end user currently is left rather uncertain about which tool to use and which quality to expect when working with new texts, and published experiences often are rather negative [16].

**Figure 1:**
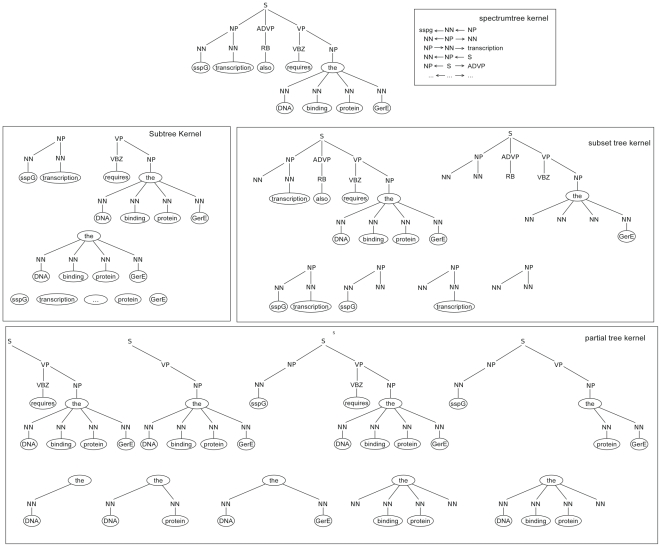
Syntax tree parse generated by the Charniak–Lease parser. The syntax tree parse of the example sentence *SsgG transcription also requires the DNA binding protein GerE*. Under the parse tree we show its substructures used by the subtree, subset tree, partial tree, and spectrum tree kernels.

**Figure 2:**
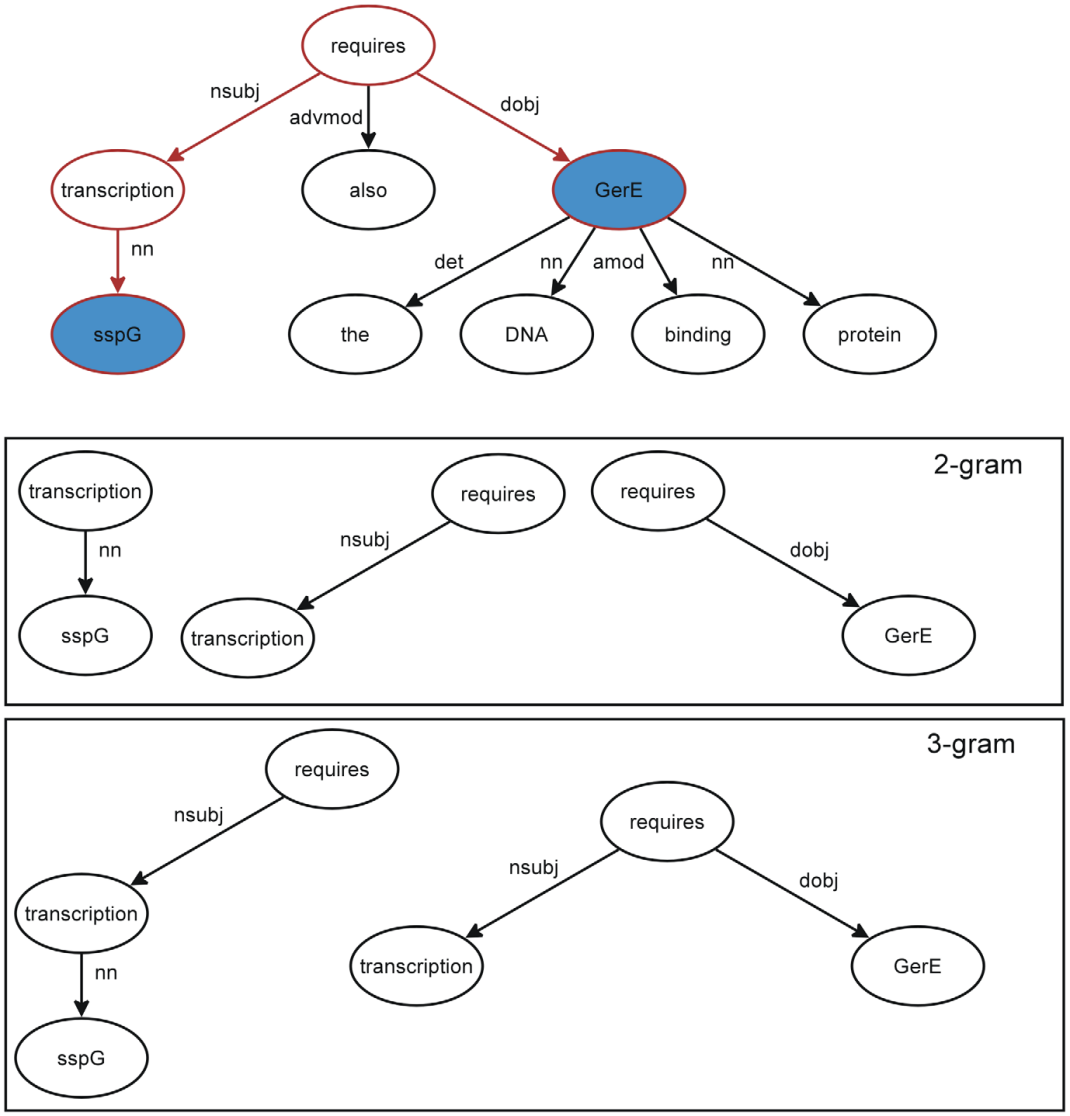
Dependency tree parse generated by the Stanford parser. The dependency tree parse of the example sentence *SsgG transcription also requires the DNA binding protein GerE*. Some substructures (paths) generated from the parse tree for kernels. We showed in red the shortest path between the two proteins (in blue), which is used by kBSPS, cosine similarity and edit distance, and all-path graphs kernels. APG kernel also uses the links outside the shortest path, but with lower weights (0.3 vs. 0.9).

In this paper, we give an unbiased and comprehensive benchmark of a large set of PPI extraction methods. We concentrate on a fairly recent class of algorithms which usually is summarized with the term *kernel methods* [17]–[33]. In a nutshell, these methods work as follows. First, they require a training corpus consisting of labeled sentences, some of which contain PPIs, some contain non-interacting proteins, and some contain only one or no protein. The exact information that later should be extracted must be known, that is, usually the pair of proteins that interact. All sentences in the training corpus are transformed into representations that try to best capture properties of how the interaction is expressed (or not for negative examples). The simplest such representation is the set of words that occur in the sentence; more complex representations are syntax trees (also called constituent trees), capturing the syntactic structure of the sentence, and dependency graphs, which represent the main grammatical entities and their relationships to each other (see Figures 1 and 2). The set of structured representations together with the PPIs are analyzed by a kernel-based learner (mostly an SVM), which learns a model of how PPIs typically are expressed. Every new sentence that should be analyzed must be turned into the same representation, which is then classified by the kernel method.

Central to the learning and the classification phases is a so-called kernel function. Simply speaking, a kernel function is a function that takes the representation of two sentences and computes their similarity. Kernel-based approaches to PPI extraction—and especially those working with *convolution kernels*—have shown high predictive accuracy and occupied top ranks in relevant CASP-style community challenges [34]. Consequently, the number of suggested methods has grown quite a bit, differing mostly in the representation they use and in the particular kernel they apply. The reported results differ largely and are difficult to compare, as often different corpora are used together with different ways of defining and measuring quality.

In this paper, we provide a comprehensive benchmark of nine kernel-based methods for relationship extraction from natural text (all substantially different approaches that were available as programs from a list of around 20 methods we considered). We tested each method in various scenarios on five different corpora. The transformation of the sentences in the corpora were performed using state-of-the-art parser software, in particular, the latest release of the Charniak–Lease parser for constituent trees and the Stanford Parser for dependency graphs. We show how publicly available kernels compare to each other in three scenarios: document-level 10-fold cross-validation (CV), cross-learning (CL), and cross-corpus (CC) settings. We also introduce a new and very fast kernel, kBSPS, and demonstrate that it is highly competitive.

We see our work as a continuation of similar benchmarks that have recently shed some light on the state-of-the-art of selected phases in the PPI extraction pipeline; in particular, these are the work on the performance of different constituent and dependency parsers [35]; on evaluation metrics and the influence of corpus properties on PPI quality [36]; an analysis of the impact of parsers on PPI performance [37]; and a recent study on the performance of different classes of features [38].

### Related Work

A number of different techniques have been proposed to solve the problem of extracting interactions between proteins in natural language text. These can be roughly sorted into one of three classes: co-occurrence, pattern matching, and machine learning. We briefly review these methods here for completeness; see [39] for a recent survey. We describe kernel-based methods in more detail in Methods.

A common baseline method for relationship extraction is to assume a relationship between each pair of entities that co-occur in the same piece of text (e.g., [36]). This “piece of text” is usually restricted to single sentences, but can also be a phrase, a paragraph, or a whole document. The underlying assumption is that whenever (two or more) entities are mentioned together, a semantic relation holds between them. However, the semantic relation does not necessarily mean that the entities interact; consequently, the kind of relation might not match what is sought. In the case of co-occurring proteins, only a fraction of sentences will discuss actual interactions between them. As an example, in the AIMed corpus (see Corpora), only 17% of all sentence-level protein pairs describe protein-protein interactions. Accordingly, precision is often low, but can be improved by additional filtering steps, such as aggregation of single PPI at the corpus level [19], removal of sentences matching certain lexico-syntactic patterns [40], or requiring the occurrence of an additional “interaction word” from a fixed list between the two proteins [15].

The second common approach is pattern matching. SUISEKI was one of the first systems to use hand-crafted regular expressions to encode phrases that typically express protein-protein interactions, using part-of-speech and word lists [41]. Overall, they found that a set of about 40 manually derived patterns yields high precision, but achieves only low recall. [42] proposed OpenDMAP, a framework for template matching, which is backed by ontological resources to represent slots and potential slot fillers, etc. With 78 hand-crafted templates, they achieve an F-score of 29% on the BioCreative 2 IPS test set [43], which was the best at the time of the competition. [44] showed that patterns can be generated automatically using manually annotated sentences that are abstracted into patterns. AliBaba goes a step further in deriving patterns from automatically generated training data [45]. The fact that automatically generated patterns usually yield high precision but low individual recall is made up by this method by generating thousands of patterns. On the BioCreative 2 IPS test set, this method achieves an F-score of around 24% without any corpus-specific tuning [45]. The third category of approaches use machine learning, for instance, Bayesian network approaches [46] or maximum-entropy-based methods [47]. The later can be set up as a two-step classification scenario, first judging sentences for relevance to discussing protein-protein interactions, and then classifying each candidate pair of proteins in such sentences. Using half of the BioCreative 1 PPI corpus each for training and testing, the approach yields an accuracy of 81.9% when using both steps, and 81.2% when using the second step only. As ML-based methods are the focus of our paper, we will discuss more closely related work in the next sections.

## Methods

In this section, we describe in detail the kernels we evaluated, the corpora and how we used them as gold standards, the measures we computed, and the parameter settings we used and how they were obtained. We believe that such a level of detail is necessary to compare different methods in a fair and unbiased manner. Note that our evaluation often produces results that are far from those published by other authors (see Results), which only underlines the importance of a clear statement regarding evaluation methods.

### Parsers

The effect of using different parsers and parse representations for the task of extracting protein-protein interactions has been investigated in [48]. In that study, the authors measured the accuracy improvements in PPI extraction when the parser output was incorporated as statistical features of the applied machine learning classifier. Their experiments showed that the investigated parsers are very similar concerning their influence on accuracy.

For our experiments we selected the Charniak–Lease re-ranking parser (ftp://ftp.cs.brown.edu/pub/nlparser/reranking-parserAug06.tar.gz) as syntax parser, since several authors [35], [49] found it as the best in recent evaluations. We used the latest official release (Aug 2006 version) with the improved self-trained biomedical model [50] using GENIA parse trees. We also performed experiments using the newer pre-release version of the same parser (courtesy of David McClosky, Eugene Charniak and Mark Johnson), and with model files trained exclusively on a news corpus and another trained on both news and PubMed abstracts [51]. However, differences in results were insignificant, and therefore we omit them for brevity. We used the Stanford conversion tool (http://nlp.stanford.edu/software/lex-parser.shtml) to obtain dependency graphs from the Charniak–Lease syntax tree parses. When explaining various kernel functions we will make use of the syntax tree (Figure 1) and the dependency tree (Figure 2) of the sentence “SsgG transcription also requires the DNA binding protein GerE,” as generated by the aforementioned parsers and tools.

### Classification with Kernels

A support vector machine (SVM) is a classifier that, given a set of training examples, finds the linear (hyper)plane that separates positive and negative examples with the largest possible margin [52]. The training examples that lie closest to the hyperplane are the support vectors. If the two sets are not linearly separable, kernel functions can transform the problem space to a nonlinear, often higher dimensional space, in which the problem might be separable [53]. The kernel is a similarity function that maps a pair of instances to their similarity score: 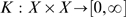, where 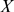 is the feature space in which the instances are represented. Given a finite set of instances, the kernel can be represented by a similarity matrix that contains all pairwise similarity scores. Kernels can be easily computed with inner products between instances without explicit feature handling that permits of the use of high dimensional feature spaces such as the rich structured representation of graphs or trees.

In our experiments we make use of SVM implementations where the training is performed by a convex quadratic programming (QP) task. Additionally, as proposed by its authors [17], the all-path graph kernel was also trained with sparse regularized least squares (RLS) [54], which requires to solve a single system of linear equations. In practice, various flavors of SVMs have been described [55]; they differ, for instance, in their training algorithm, parameter set, or representation of features. Furthermore, several freely available implementations exist, among which SVM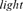 [52] and LIBSVM [56] probably are the most renowned ones. Both can be adapted to special needs—such as working with linguistic structures—by providing an option to integrate user-defined kernel functions. There are two alternatives for the integration of a kernel functions. In SVM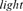 one can code his own kernel function that accepts the corresponding instance representation (with option -t 4 and a self-implemented kernel.h). LIBSVM supports the use of pre-computed kernels, i.e., the kernel function is passed to the SVM learner as a Gram-matrix, containing the pairwise similarity of all instances. Most of the kernels we experimented with use the SVM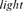 implementation, except for the shallow linguistic kernel that uses LIBSVM. To be able to measure the AUC score (see Evaluation Methods) we had to apply changes in the LIBSVM code to retrieve not just the class label, but also the value of the prediction.

### Kernel Methods

The kernels introduced in this section are mostly convolution kernels [57], i.e., they make use of the structure of the instances (in our case, syntax trees or dependency graphs of sentences). Their main idea is to quantify the similarity of two instances through counting the similarities of their substructures; however, there have been many proposals on to how to do this in the best way. We include into our experiments all publicly available approaches that make use of different kernel functions we are aware of. We were able to obtain nine out of the about 20 considered kernels (see Tables S1 and S2), either from a publicly available download, or upon request from the respective authors (details about kernel packages are in Table S3, all software source code are available with installation instruction on our website http://informatik.hu-berlin.de/forschung/gebiete/wbi/ppi-benchmark).

Most of these kernels have been specifically designed to extract PPI from text or have been successfully applied to this task. Exceptions are subtree, partial tree and spectrum tree kernels which to our knowledge were not tested for PPI extraction before. Next, we will very briefly introduce their underlying principles (see also Table S1 for an overview).

#### Shallow linguistic kernel (SL)

From all kernels we tested, this is the only one that exclusively uses shallow parsing information [23]. We included it to contrast its performance from the more complex convolution kernels. The kernel is defined as the sum of two kernels, the global and the local context kernels. The feature set of the *global context kernel* is based on the words occurring in the sentence *fore-between*, *between* and *between-after* relative to the pair of investigated proteins. Based on this, three term frequency vectors are created according to the bag-of-words paradigm. The global kernel is then obtained as the count of common words in the three vectors obtained from the two compared sentences. The *local context kernel* uses surface (capitalization, punctuation, numerals) and shallow linguistic (POS-tag, lemma) features generated from tokens left and right to proteins of the protein pair (the size of the window is adjustable). The similarity of the generated pairs of left and right feature vectors is calculated using scalar product.

#### Subtree kernel (ST)

The next four kernels use the syntax tree representation of sentences (see Figure 1). They differ in the definition of extracted substructures. The *subtree kernel* considers all common subtrees in the syntax tree representation of two compared sentences [30]. Therein, a subtree is a node with all its descendants in the tree (see again Figure 1). Two subtrees are identical if the node labels and order of children are identical for all nodes.

#### Subset tree kernel (SST)

The *subset tree kernel* relaxes the constraint that all descendants, including leaves, must always be included in the substructures [20]. It retains the constraint that grammatical rules must not be broken. For a given tree node, either none or all of its children must be included in the resulting subset tree (see Figure 1). As for the ST kernel, the order of child nodes matters.

#### Partial tree kernel (PT)

The *partial tree kernel* is the most permissive syntax-tree-based kernel we considered [28]. It allows virtually any tree substructures; the only constraint that is kept is that the order of child nodes must be identical (see Figure 1).

#### Spectrum tree kernel (SpT)

The *spectrum tree kernel* focuses on simpler syntax-tree substructures than those discussed so far. It compares all vertex-walks (v-walks), sequences of edge-connected syntax tree nodes, of length 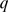 (also known as 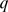-grams, [58]). Note that the orientation of edges is important: the vertex-walks 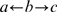 and 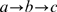 are thus not identical (see Figure 1).

#### 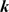-band shortest path spectrum kernel (kBSPS)

**Figure 3:**
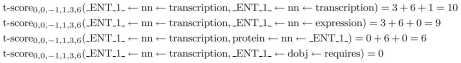
Examples of tolerant matching. L, E mismatches are tolerated (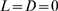), D mismatches are untolerated (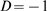); similarity weights are 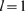, 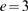, 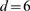. For kBSPS, we use default values 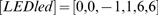, 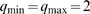, and 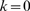 for the kernel.

In [29], we proposed a new kernel function that is an extension of the SpT kernel. As it was not published before, we explain it here in more detail. kBSPS combines three ideas: First, the syntax-tree-based SpT kernel is adapted to dependency graphs. Second, the definition of v-walk is extended and when comparing two v-walks, certain mismatches are allowed. Third, it considers not only the shortest path between two proteins in the graph (as many others do), but also adds neighboring nodes. The first two extensions work as follows. The kBSPS kernel first includes edge labels into v-walks, which determine the dependency type of a relationship (see Figure 2). For consistency, the length of such v-walks remains the number of included nodes, i.e., edges are not counted into the length. Vertex-walks of dependency graphs contain on average more surface tokens than syntax tree v-walks, because the latter contain surface tokens only in leaves, of which at most two may be present in any syntax tree v-walk. Since the variation in surface tokens is much larger than in internal nodes of syntax trees, a tolerant matching is necessary to allow for linguistic variation. This tolerant matching distinguishes three types of nodes: dependency types (D), candidate entities (E), and other surface tokens (L). Mismatches/matches are then scored differently depending on the type of nodes (determined by appropriate parameters). When two v-walks are compared, a tolerated mismatch assigns score 0 only to the given node in the v-walk, while an untolerated mismatch sets the entire similarity score to 0 (see examples in Figure 3). The third extension changes the substructures that are compared by representing them as v-walks. Instead of using all v-walks of the dependency graph, kBSPS starts from only considering those one lying on the shortest path between the investigated entity pair. It is widely acknowledged that tokens on this path carry most information regarding their relationship; however, in some cases, interacting words are outside this scope, like in “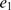 is an 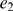 binding protein.” Therefore, optionally, kBSPS also adds all nodes within distance 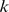 from the shortest path of the investigated entity pair. The resulting subgraph is called 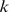-band shortest path of a pair 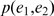. Finally, the similarity of two entity pairs 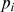 and 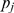 is calculated as:
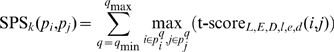
(1)where 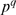 is the set of v-walks of length 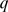 generated from the 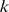-band shortest path of pair 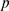, 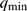 and 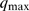 control the range of 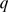, and 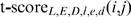 is the tolerant matching score (defined in Supporting Information, Text S1, exemplified in Figure 3).

#### Cosine similarity kernel (cosine)

In [22], the authors define two kernel functions based on the cosine similarity and the edit distance among the shortest paths between protein names in a dependency tree parse (see Figure 2). Let 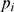 and 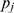 be two such shortest paths between two pairs of analyzed entities. The *cosine similarity kernel* calculates the angle between the representation of 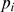 and 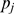 as vectors of term frequencies in a vector space. Basically cosine counts the number of common terms of the two paths, normalized by the length of the paths.

#### Edit distance kernel (edit)

The drawback of the cosine similarity for textual data is its order-independence. The *edit distance kernel*, also proposed in [22], overcomes this issue. Therein, the distance between two paths is defined as the edit distance between them, i.e., the minimal number of operations (deletion, insertion, substitution at word level) needed to transform one path into the other, normalized by the length of the longer path. This measure is converted into a similarity measure using:
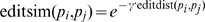
(2)where 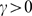 is a parameter.

#### All-paths graph kernel (APG)

The *all-paths graph kernel* [17] counts weighted shared paths of all possible lengths. Paths are generated both from the dependency parse and from the surface word sequence of the sentence. Path weights are determined by dependencies weights which are the higher the shorter the distance of the dependency to the shortest path between the candidate entities is. One peculiarity of Airola's method is the usage of the sparse regularized least squares (RLS) method (instead of standard SVM), which is a state-of-the-art kernel-based machine learning method that scales very well with very large training sets. For comparison, we also trained APG kernel with SVM.

#### Other kernels

In the literature, several further kernel-based approaches to relationship extraction were proposed. We give a brief survey of them below. Note that most of these kernels are either unavailable as programs or very similar to at least one of those we selected for our benchmark (see also Table S2).

In [25] predicate, walk, dependency, and hybrid kernels are proposed, each operating on dependency trees extended with shallow linguistic and gazetteer information. The *walk kernel* showed the best performance. It generates vertex-walks and edge-walks (edge-based counterpart of v-walks) of fixed length two on syntactic (POS) and lexical (token) level along the shortest path between the analyzed entities. A polynomial SVM kernel was applied to calculate the similarity between vectors. The idea of Kim was developed further in [26] by augmenting the original feature set with additional sentence characteristics, for example, word stems of all tokens and shortest path length. Since the feature set can get pretty large (10k+ features), feature selection is applied before training. Both kernels were unavailable.

In [21], a kernel that used subtrees of dependency trees is proposed. The nodes of the dependency tree were augmented with various syntactic and semantic features. A kernel function was applied to compare subtrees, calculating the common contiguous or sparse subsequences of nodes, which incorporated a similarity function for the augmented features. A similar kernel function was proposed in [32], albeit with a smaller feature set. The source code for these kernels is not publicly available.

The general sparse subsequence kernel for relation extraction [19] calculates the total number of weighted subsequences of a given length between two strings. Sentences are represented by fore-between, between, and between-after sequences relative to the investigated entity pair. The sequences can be defined over various alphabets, such as set of words, POS tags, or broader word classes. This kernel is similar to SL kernel proposed in [23]. SL kernel uses similar feature sets and it is computationally much more effective, though order-independent kernel.

A mixture of previous approaches was proposed in [31], called *convolution dependency path kernel*, which combined the beneficial high recall of subsequence kernels with the reduced feature space using syntactic information of shortest path of dependency trees. The combined kernel applied the subsequence kernel on the shortest dependency paths, which makes it very similar to the method of [21]. The source code of the kernel was not available.

In [27], a combined multiple layers of syntactic information is proposed. A bag-of-words kernel, a subset tree kernel [28], and an APG kernel [17] were used together with dependency parses and deep parses. The kernels were combined simply through summing the normalized values of each kernel for each parse. The hybrid kernel is currently not available.

In [24], the authors used the Smith–Waterman distance function when comparing two string sequences. Their *local alignment kernel* was then defined as the sum of SW scores on all possible alignments between the strings. To compute SW distance, a substitution matrix should be initialized with the pairwise similarity of any two words. The matrix elements were estimated by distributional similarity measures calculated on a large independent corpus, which is very costly in terms of time. The source of the approach is currently not available.

### Corpora

There is no widely accepted definition of the concept of PPI, i.e., what should be annotated as PPI in text, therefore methods evaluated on different PPI-annotated corpora are difficult to compare. In [36], a thorough analysis of five freely available PPI-annotated resources, namely AIMed [18], BioInfer [59], HPRD50 [60], IEPA [61], and LLL [62], was performed. Some basic statistics of the corpora can be found in Table 1. Although all of these corpora carry information about named entities and all annotate PPIs, there are many aspects in which the corpora show significant differences. Corpora differ in quite a few aspects, for instance, the scope of annotated entities varies (typically proteins and genes, some also RNAs, but IEPA only chemicals), the coverage of entities is not always complete, some corpora specify the direction of interactions, just to name a few. As “greatest common factor” among the notions of PPI, in [36] it is suggested to use only the information on undirected, untyped interactions (among a few other constraints) for evaluation purposes. We also followed this suggestion.

**Table 1:** Basic statistics of the 5 corpora used for kernel evaluation.

Corpus	Sentences	Positive pairs	Negative pairs
AIMed	1955	1000	4834
BioInfer	1100	2534	7132
HPRD50	145	163	270
IEPA	486	335	482
LLL	77	164	166

Pairs are checked for (orderless) uniqueness; self-interacting proteins are excluded.

**Figure 4:**
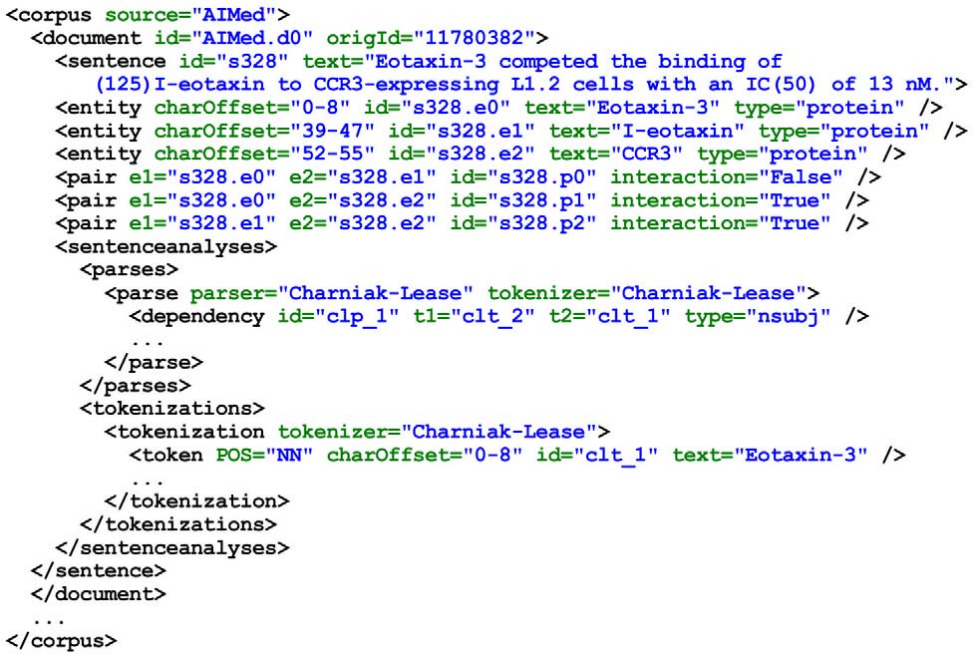
The general structure of the learning format.

In the same study, an XML-based format was also defined for annotating PPIs, called *PPI learning format*. The authors transformed all five aforementioned corpora into this format, which we reuse. The general structure of the learning format is shown in Figure 4. Each corpus consists of documents, and documents consist of sentences. The sentence text is located in the attribute *text*. The actual annotation of named entities and their relations is encoded through *entity* and *pair* elements. The position of an entity in the sentence text is specified in the *charOffset* attribute.

The presence or absence of a relation is marked on the level of named entity pairs, not on the level of sentences (cf. attribute *interaction* of *pair* in Figure 4), which enables the annotation of multiple entity pairs per sentence. For instance, in the sentence in Figure 4, there is a relation between entities 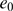 and 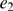 and 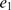 and 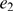, whereas there is no relation between entities 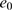 and 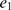. Consequently, the learning examples used by a classifier correspond to entity pairs rather than to sentences. The learning format also provides means for expressing token boundaries and dependency parses of sentences, and it allows to store several alternative tokenizations and parses for a given sentence.

### Evaluation Methods

We use various performance measures to evaluate kernel-based classifiers for PPI extraction. On one hand, we report on the standard evaluation measures: precision, recall, and F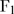-score. F-score has been criticized recently as inadequate for PPI extraction because of its sensitivity to the ratio of positive/negative examples in the training set [17], [36]. Therefore, we also report on the AUC measure (area under the receiver operating characteristics curve) of the methods, which is invariant to the class distribution in the data sets. We evaluated all kernel methods in three different settings: Cross-validation, cross-learning, and cross-corpus. None of these is new; cross-validation still seems to be the current de facto standard in PPI extraction, cross-learning was proposed in [26], and cross-corpus was, for instance, used in [15], [17], [63].

#### Cross-Validation (CV)

In this setting, we train and test each kernel on the same corpus using document-level 10-fold cross-validation. We refrain from using the also frequently mentioned instance-level splitting, in which every sentence containing more than two protein names may appear, though with different labeling, both in the training and the test sets. This is a clear case of information leakage and compromises the evaluation results. Its impact on PPI results is higher than in many other domains, since in PPI corpora sentences very often contain more than two protein names. We employ the document-level splits that were used by Airola and many others, which allow direct comparison of the results. We indicate the standard deviation of the averaged 10-fold cross-validation values.

#### Cross-Learning (CL)

Although the document-level 10-fold cross-validation became the de facto standard of PPI relation extraction evaluation, it is also somewhat biased, because the training and the test data sets have very similar corpus characteristics. It was shown [36] that the different positive/negative interaction pair distribution of the five benchmark corpora accounts for a substantial part of the diversity of the performance of approaches. Since the ultimate goal of PPI extraction is the identification of PPIs in biomedical texts with unknown characteristics, we performed experiments with learning across corpora, where the training and test data sets are drawn from different distributions. In CL experiments, we train on the ensemble of four corpora and test on the fifth one.

#### Cross-Corpus (CC)

Finally, in CC experiments, we train the model on one corpus and then test on the other four corpora.

Apart from measuring the quality of the extractions, we also looked at the time it takes to classify the corpora. Whenever the texts to be analyzed are large, classification time may be the decisive factor to choose a method. However, we did not take particular measures to obtain perfect run times (eliminating all concurrent processes on the machines), so our times should only be considered as rough estimates. We should also mention that all the tested software are prototypes where the efficiency of implementations may significantly differ. Nevertheless, these figures should be good indicators of what can be expected when using the kernels out-of-the-box. Note that all methods we analyzed also require extra time (in addition to classification) to parse sentences.

### Experimental Setup

#### Entity blinding

All corpora we use for evaluation have all entities readily annotated. This means that our results only measure the performance of PPI extraction and are not influenced by problems of named entity recognition. However, to produce the right format for the kernel methods, we apply entity blinding, that is, we replace named entity occurrences with a generic string. Entity blinding is usually applied in relation extraction systems to (1) inform the classifier about the location of the NEs; (2) ensure the generality of the learned model, since classifiers should work for any entity in the given context. Before doing that we had to resolve the entity–token mismatch problem.

**Figure 5:**
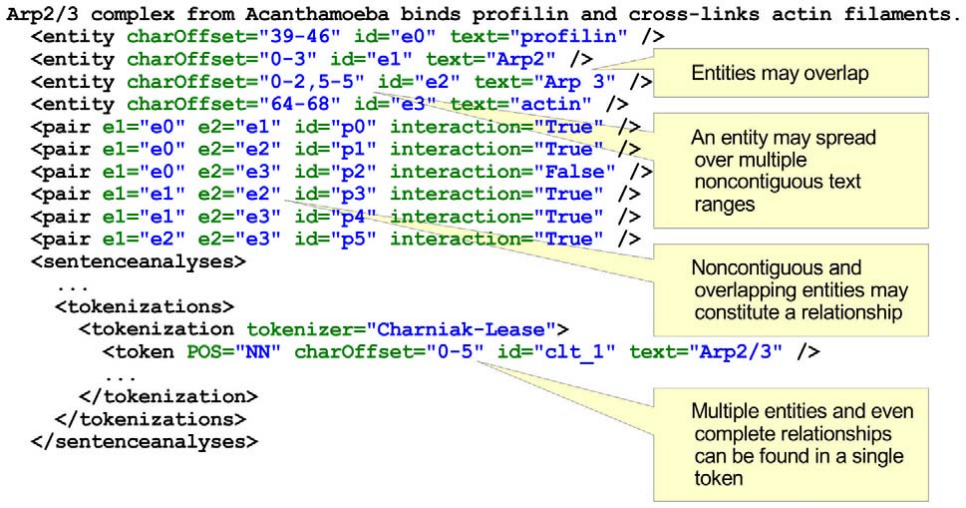
Learning format pitfalls (sentence BioInfer.d77.s0). (1) Named entities may overlap. The string Arp2/3 contains two named entities, namely Arp2 and Arp3. (2) An entity may spread over multiple noncontiguous text ranges. The entity Arp3 paragraph spreads over two ranges [0–2] and [5–5]. (3) Such noncontiguous and overlapping entities may constitute a relation, such as in The Arp2/3 complex….

Syntax and dependency parsers work on token-based representation of sentence text being the output of the tokenization, also encoded in the learning format. Entities, however, may not match directly contiguous token sequences; this phenomenon has to be resolved for enabling the entity-based referencing of PPIs. Practically all combinations of entailment and overlapping occur in text: one entity may spread over several tokens or correspond merely to a part of a token, and there may exist several named entities in one token. We depicted some examples of the entity–token mismatch phenomenon in Figure 5.

In order to overcome these difficulties and adopt a clear entity–token mapping concept, we apply the following strategy: every token that at least partly overlaps with an entity is marked as entity. Entity blinding is performed as follows: A sentence with 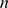 entities contains 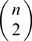 possibly differently labeled entity pairs (see Figures 4+5). For each entity pair of the sentence, we replicate the sentence and create a separate learning example. In order to distinguish entities of the learning example from other entities, we label all tokens of the entity pair under consideration as _ENT_1_ and _ENT_2_, respectively, while we label the others as _ENT_. In case of overlapping entities (cf. Figure 5), we use the special label _ENT_1_AND_2_ for the token including both entities; this strategy was also applied in [17].

#### Constituent tree parses

Since some of the selected kernel methods, namely ST, SST, PT and SpT kernels are defined for syntax trees, we injected the syntax tree parses into the learning format. The terminal symbols of the syntax tree parses (i.e., tokens) were mapped to the character offsets of the original sentence text. This was necessary for the entity blinding in the constituent tree parse. Finally, the parses were formatted so that they comply with the expectations of the given kernel's implementation (the extended corpus files are available at our web site).

#### Parameter optimization

All evaluated methods have several parameters whose setting has significant impact on the performance. To achieve best results, authors often apply an exhaustive systematic parameter search—a multidimensional fine-grained grid search for myriads of parameter combinations—for each corpus they evaluate on. However, results obtained in this way cannot be expected to be the same as for other corpora or for new texts, where such an optimization is not possible. In this study, we take the role of an end-user which has a completely new text and wants to choose a PPI extraction method to extract all PPIs from this text. Which parameters should this user apply?

Ideally, one could simply use the default parameters of the kernels, leaving the choice of best settings to the authors of the kernels. This was our initial idea, which we had to abandon for two reasons: (1) for some syntax-tree-based kernels (ST, SST, PT), the default regularization parameter of the learner, 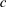, often produced 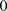
% F-score; (2) for APG there is no explicit default parameterization. As a compromise, we resorted to a coarse-grained grid parameter search only on a small set of important parameters (see Table S4). We selected the best average setting as the *de facto* default setting for each kernel. We did not perform separate optimization runs for AUC and F-score, thus reported data always belong to the same experiment.

Also in CC evaluation, optimization geared towards the test-corpus may improve the performance. As shown in [17, Tables 3 and 4], the F-score can raise tremendously (sometimes by 50 points) when the APG-based classifier is optimized with a threshold according to the ratio of positive/negative pairs in the test corpus. We refrained from using such an optimization technique at CC evaluation, because again such information is not available in a real world application.

## Results

We performed a thorough evaluation of nine different methods for extracting protein-protein interactions from text on five different, publicly available and manually annotated corpora. All methods we studied classify each pair of proteins in a sentence using a kernel function. The methods differ widely in their individual definition of this kernel function (comparing all subtrees, all subsets, all paths,…), use different classifiers, and make use of different types of information (shallow linguistic information, syntax trees or dependency graphs).

We report results in three different scenarios. In *cross-validation*, each corpus is treated independently from each other. Reported results are the average over a document-level 10-fold cross-validation per corpus. Even though this strategy is the de facto way of evaluating PPI extraction systems, its results cannot be safely extrapolated to the application of a method on completely new text, as the model that is learned overfits to the particular corpus. In *cross-learning*, training and test data come from different corpora altogether. We report results on five experiments, where in each experiment each method was trained on four corpora and tested on the fifth. This strategy should produce results that are much more likely to hold also on unseen texts. A variation of this strategy is *cross-corpus*, where we always train on one corpus and evaluate on the other four. Obviously, one expects worse results in CC than in CL, as the diversity of training data is reduced, while the heterogeneity in the test data is increased.

### Cross-Validation

**Figure 6:**
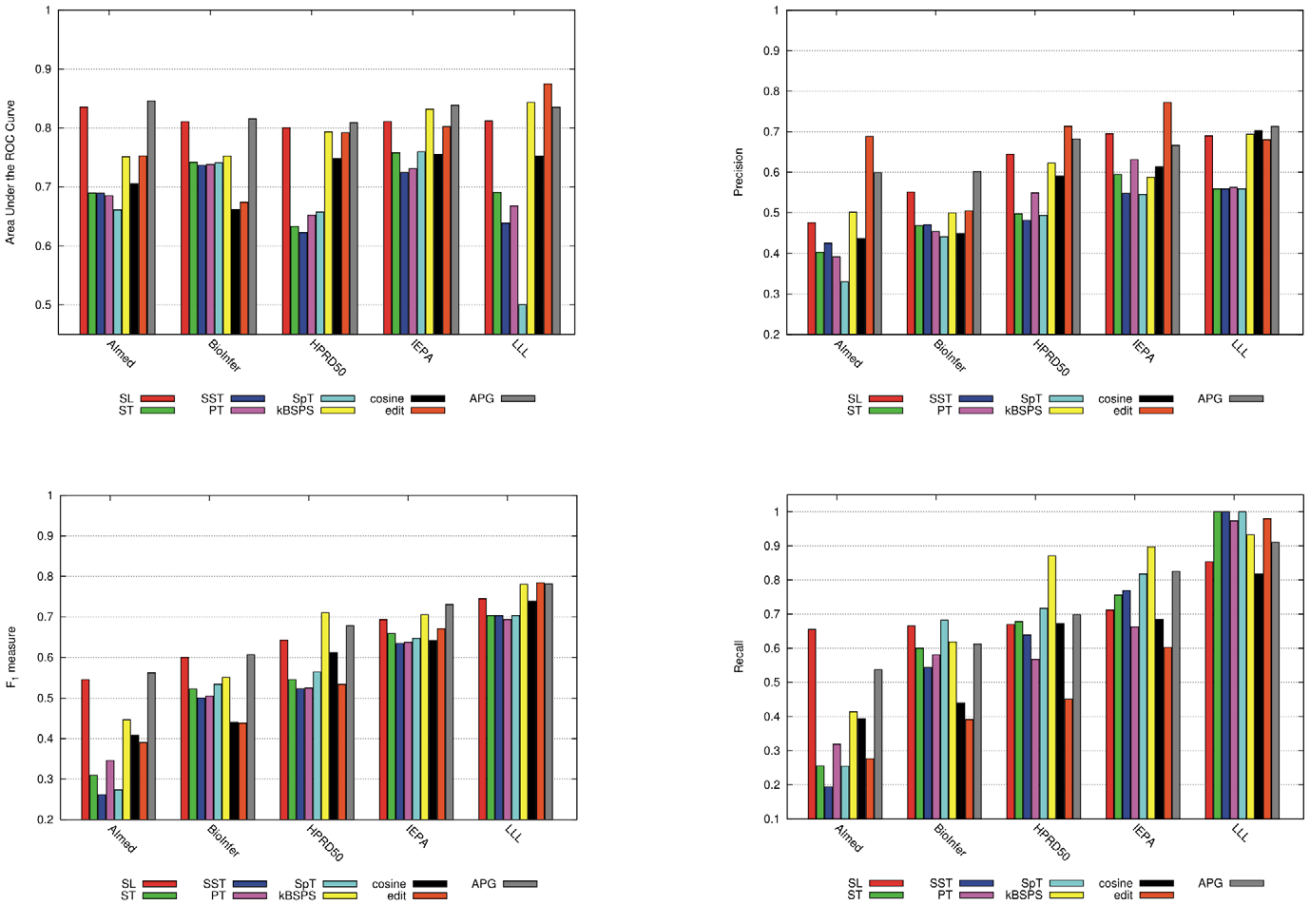
AUC, F-score, precision and recall values with CV evaluation, including standard deviation measured on the 10 folds.

Table 2 and Figure 6 give results of CV on a per-corpus basis. In the table, for SL, kBSPS, cosine, edit, and APG kernels we provide both our own measurements and the ones published in the respective original paper. We also ran APG with SVM. Recall that, to closely imitate the real word scenario, we did not perform a systematic parameter tuning (see Methods). Table 2 also contains results for rich-feature-vector-based kernel [26] and hybrid kernel [63], which are both not covered in our evaluation. As a baseline, we additionally give precision/recall/F-score values for the sentence based co-occurrence methods and the rule-based RelEx [60].

**Table 2:** 10-fold document-level CV results.

Kernel	AIMed				BioInfer				HPRD50				IEPA				LLL			
	AUC	P	R	F	AUC	P	R	F	AUC	P	R	F	AUC	P	R	F	AUC	P	R	F
SL	83.5	47.5	**65.5**	54.5	**81.1**	55.1	66.5	**60.0**	**80.0**	64.4	67.0	64.2	81.1	69.5	71.2	69.3	81.2	69.0	85.3	74.5
ST	68.9	40.3	25.5	30.9	74.2	46.8	60.0	52.2	63.3	49.7	67.8	54.5	75.8	59.4	75.6	65.9	69.0	55.9	**100.**	70.3
SST	68.9	42.6	19.4	26.2	73.6	47.0	54.3	50.1	62.2	48.1	63.8	52.2	72.4	54.8	76.9	63.4	63.8	55.9	**100.**	70.3
PT	68.5	39.2	31.9	34.6	73.8	45.3	58.1	50.5	65.2	54.9	56.7	52.4	73.1	63.1	66.3	63.8	66.7	56.2	97.3	69.3
SpT	66.1	33.0	25.5	27.3	74.1	44.0	**68.2**	53.4	65.7	49.3	71.7	56.4	75.9	54.5	81.8	64.7	50.0	55.9	**100.**	70.3
kBSPS	75.1	50.1	41.4	44.6	75.2	49.9	61.8	55.1	79.3	62.2	**87.1**	**71.0**	**83.2**	58.8	**89.7**	70.5	84.3	69.3	93.2	**78.1**
cosine	70.5	43.6	39.4	40.9	66.1	44.8	44.0	44.1	74.8	59.0	67.2	61.2	75.5	61.3	68.4	64.1	75.2	70.2	81.7	73.8
edit	75.2	**68.8**	27.7	39.0	67.4	50.4	39.2	43.8	79.2	71.3	45.2	53.3	80.2	**77.2**	60.2	67.1	**87.5**	68.0	98.0	**78.4**
APG	**84.6**	59.9	53.6	**56.2**	**81.5**	**60.2**	61.3	**60.7**	**80.9**	**68.2**	69.8	67.8	**83.9**	66.6	82.6	**73.1**	83.5	**71.3**	91	**78.1**
APG (with SVM)	71.2	62.9	48.9	54.7	73.9	**60.2**	63.4	**61.6**	74.1	65.4	72.5	67.5	76.2	71.0	75.1	72.1	74.9	**70.9**	95.4	**79.7**
SL [23]		60.9	57.2	59.0																
kBSPS [29]	67.2	49.4	44.7	46.1					76.9	66.7	80.2	70.9	75.8	70.4	73.0	70.8	78.5	76.8	91.8	82.2
cosine [22] †		62.0	55.0	58.1																
edit [22] †		77.5	43.5	55.6																
APG [17]	84.8	52.9	61.8	56.4	81.9	56.7	67.2	61.3	79.7	64.3	65.8	63.4	85.1	69.6	82.7	75.1	83.4	72.5	82.2	76.8
rich-feature-based [26]		49.0	44.0	46.0						60.0	51.0	55.0		64.0	70.0	67.0		72.0	73.0	73.0
hybrid [63]	86.8	55.0	68.8	60.8	85.9	65.7	71.1	68.1	82.2	68.5	76.1	70.9	84.4	67.5	78.6	71.7	86.3	77.6	86.0	80.1
co-occ. [17]		17.8	100.	30.1		26.6	100.	41.7		38.9	100.	55.4		40.8	100.	57.6		55.9	100.	70.3
RelEx [36]		40.0	50.0	44.0		39.0	45.0	41.0		76.0	64.0	69.0		74.0	61.0	67.0		82.0	72.0	77.0

The first two blocks contain the results of our evaluation, the third block contains corresponding results of kernel approaches from the literature, and the third block shows some non-kernel-based baselines. Bold typeface shows our best results for a particular corpus (differences under 1 base point are ignored). AUC, precision, recall, and F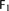-score in percent.

† instance-level CV.

Table 2 shows that we often could not reproduce results reported by the authors. However, we want to emphasize that our study is the first to provide an unbiased comparison of different methods where each method was presented exactly the same training and test data and where the same tuning procedures were used (see Methods). The differences may have different reasons. First, evaluation strategies differ (different splits or document- vs. instance-level CV). Second, parameter tuning was different. Third, corpora were treated differently. We provide examples below.

In case of the AIMed corpus, there are different interpretations regarding the number of interacting and non-interacting pairs [64]. The learning format we applied contains 1000 positive and 4834 negative examples (cf. Table 1), while in [23] (SL kernel) 8 more positive and 200 fewer negative examples are reported. If the entity blinding is performed only partially, that can also affect the performance of the learner. Using the same learning format as in our paper, with the shallow linguistic kernel of [23] an F-score of 52.4% was achieved, which is actually somewhat worse than our result of 54.5%.

In case of the cosine and the edit kernels, the figures reported in the original paper were achieved with instance-level CV (personal communication, not mentioned in the original paper). As noted earlier in the literature [17], [64], this strategy increases F-score significantly (on AIMed by 18%) but relies on information leakage.

We account for smaller differences in F-score to the fact that we used different parameter optimization than in the original works. This is, for instance, the case for kBSPS (our own implementation) and APG. However, recall that parameter tuning always carries the danger of overfitting to the training data. The relative performance of different kernels in our results should be fairly robust due to the usage of the same tuning strategy for all kernels, while better results can be achieved by performing further corpus-specific tuning. Interestingly, for APG, we obtained better F-score and AUC values than the published ones for two of the five corpora.

Based on the results in Table 2, we can roughly divide the kernels into three groups. Syntax-tree-based kernels (ST, SST, PT, SpT) oftentimes are just on par with the co-occurrence approach in terms of F-score. They are clearly better than co-occurrence only on BioInfer and IEPA. On the very small LLL, their results practically coincide with co-occurrence. The second group consists of cosine and edit. These two usually outperform co-occurrence (in some cases significantly), but their performance does not exceed the one of the rule-based RelEx method in terms of F-score. The cosine kernel on average delivers better F-scores, while the edit kernel gives higher AUC values. Both of the former groups are outperformed by APG, SL and kBSPS. The figures show that there is only an insignificant difference among APG and SL on the more important larger corpora (AIMed and BioInfer), while on the three smaller ones (HPRD50, IEPA, and LLL) SL has slightly lower scores when compared to APG and kBSPS kernels. Note that when APG is trained with SVM its AUC score drops below average (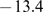 on AIMed and 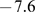 on BioInfer) while its F-score remains among the best. These three kernels clearly outperform the rule-based RelEx on AIMed and BioInfer, and are slightly better on average on the other corpora.

### Cross-Learning

**Figure 7:**
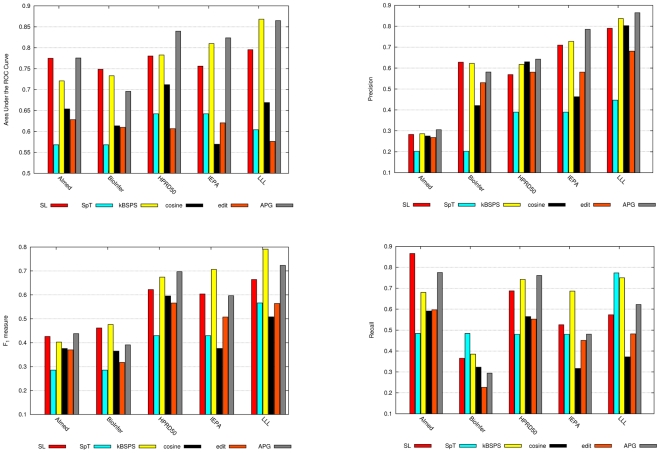
AUC, F-score, precision and recall values with CL evaluation.

Table 3 and Figure 7 show our results for CL performance. Because the training on the ensemble of four corpora generally takes much longer time, we computed results only for the fastest out of the four syntax-tree-based kernels (SpT), since all of them performed similarly low in the CV setting. This trend is confirmed, as SpT also here performs considerably worse than all other tested methods. We also looked for CL results in the literature. Beside the results of the combined kernel proposed in [25] (numbers showed in the table are taken from [38]), the only one we could find were produced without the BioInfer corpus [26]. This means that classifiers were trained only on three corpora. Since BioInfer contains the largest number of entity pairs, these numbers are not directly comparable to ours and therefore omitted.

**Table 3:** Cross-learning results.

Kernel	AIMed				BioInfer				HPRD50				IEPA				LLL			
	AUC	P	R	F	AUC	P	R	F	AUC	P	R	F	AUC	P	R	F	AUC	P	R	F
SL	**77.5**	28.3	**86.6**	42.6	**74.9**	62.8	36.5	46.2	78.0	56.9	68.7	62.2	75.6	71.0	52.5	60.4	79.5	79.0	57.3	66.4
SpT	56.8	20.3	48.4	28.6	64.2	38.9	**48.0**	43.0	60.4	44.7	**77.3**	56.6	54.2	41.6	19.6	15.5	50.5	48.2	**83.5**	61.2
kBSPS	72.1	28.6	68.0	40.3	73.3	62.2	38.5	**47.6**	78.3	61.7	74.2	67.4	81.0	72.8	**68.7**	**70.7**	**86.8**	83.7	75.0	**79.1**
cosine	65.4	27.5	59.1	37.6	61.3	42.1	32.2	36.5	71.2	63.0	56.4	59.6	57.0	46.3	31.6	37.6	66.9	80.3	37.2	50.8
edit	62.8	26.8	59.7	37.0	61.0	53.0	22.7	31.7	60.7	58.1	55.2	56.6	62.1	58.1	45.1	50.8	57.6	68.1	48.2	56.4
APG	**77.6**	**30.5**	77.5	**43.8**	69.6	58.1	29.4	39.1	**84.0**	**64.2**	76.1	**69.7**	**82.4**	**78.5**	48.1	59.6	**86.5**	**86.4**	62.2	72.3
Fayruzov *et al.*	72.0			40.0	70.0			31.0	75.0			56.0	68.0			29.0	74.0			39.0

Classifiers are trained on the ensemble of four corpora and tested on the fifth one. Rows correspond to test corpora. Best results are typeset in bold (differences under 1 base point are ignored). We show for reference the results with the combined full kernel of [25], taken from [38]. AUC, precision, recall, and F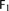-score in percent.

The overall trend from CV to CL confirms our expectation. Performance results drop significantly, sometimes by more than 15 points. The most stable is the kBSPS kernel (average drop AUC: 1.12, F: 2.84); in a few cases CL outperforms CV results (also seen with APG on HPRD). The SL and APG kernels show a modest drop in AUC (4.5 and 2.82), which gets larger by F-score (9.28 and 10.22). Cosine and edit suffer from the most significant drops.

We can form two groups of kernels based on their CL performance. The first consists of SpT, cosine, and edit—supposedly other syntax-tree-based kernels belong here as well. SpT is clearly the worst in this comparison. Two outlier corpora are BioInfer and IEPA: on the former SpT is on par with other kernels, while on the latter it achieves very low value due to the extremely low recall. Cosine and edit are just somewhat better than SpT, particularly on AIMed and IEPA. Their AUC scores are mostly just above 60%, and their F-scores outperform the co-occurrence methods only on AIMed. On IEPA and LLL, all three F-scores are inferior to the co-occurrence baseline.

The other group consists of SL, kBSPS, and APG kernels. The SL kernel produced the least divergent values across the five corpora in terms of both major evaluation measures. It shows performance comparable with the best kernels on the two larger corpora, but is somewhat inferior on the three smaller ones. The AUC values of our kBSPS kernel are improved with decreasing size of the test corpus, and are comparable on most corpora with the SL and APG kernel, except for AIMed (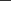
5%). For F-scores, the size dependent tendency is somewhat similar, but here the kBSPS kernel outperforms the other kernels on three corpora, with a remarkable margin of 8–10% on IEPA and LLL. APG results are comparable or better for AUC than the ones of kBSPS and SL kernel, except on BioInfer. It achieved the best F-score value on AIMed and HPRD50, but on the other three corpora its performance is clearly below kBSPS.

Finally, it is interesting to compare the performance of the better group with RelEx, the rule-based baseline (which requires no learning at all). We can see that on most corpora, only the best kernel-based method is comparable with RelEx, and except on BioInfer, the difference is a mere few percent.

### Cross-Corpus Evaluation

**Figure 8:**
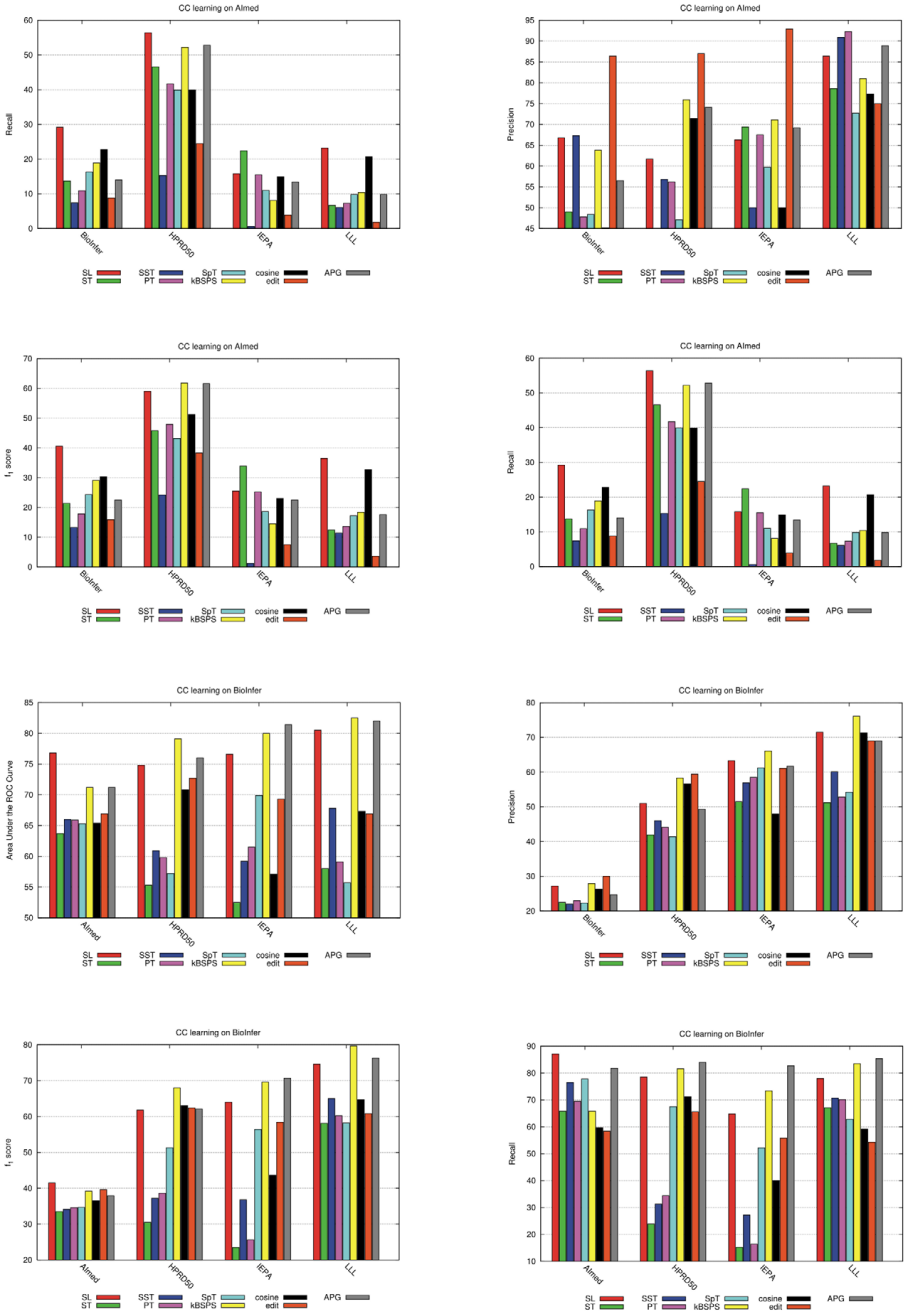
AUC, F-score, precision and recall values with CC evaluation trained on AIMed and BioInfer.

Table 4 and Figure 8 show cross-corpus results for classifiers trained on AIMed and BioInfer for some selected kernels. Results for all other kernels and for classifiers trained on HPRD50, IEPA and LLL can be found in Table S5.

**Table 4:** Cross-corpus results trained on AIMed and BioInfer.

Kernel	Training corpus	AIMed				BioInfer				HPRD50				IEPA				LLL			
		AUC	P	R	F	AUC	P	R	F	AUC	P	R	F	AUC	P	R	F	AUC	P	R	F
SL	AIMed	(83.5)	(47.5)	(65.5)	(54.5)	**73.1**	66.8	**29.2**	**40.6**	72.9	61.7	56.4	59.0	68.8	66.3	15.8	25.5	72.6	86.4	23.2	36.5
	BioInfer	**76.8**	27.2	**87.1**	**41.5**	(81.1)	(55.1)	(66.5)	(60.0)	74.8	51.0	78.5	61.8	76.6	63.3	64.8	64.0	80.5	71.5	78.0	74.6
SpT	AIMed	(66.1)	(33.0)	(25.5)	(27.3)	69.5	48.4	16.3	24.3	60.0	47.1	39.9	43.2	67.9	59.7	11.0	18.6	57.0	72.7	29.8	17.2
	BioInfer	65.3	22.3	77.8	34.7	(74.1)	(44.0)	(68.2)	(53.4)	57.2	41.4	67.5	51.3	69.9	61.2	52.2	56.4	55.7	54.2	62.8	58.2
kBSPS	AIMed	(75.1)	(50.1)	(41.4)	(44.6)	69.9	71.6	15.0	24.8	76.8	77.5	38.0	51.0	73.6	66.7	25.4	29.9	75.1	85.7	27.3	13.5
	BioInfer	71.8	**29.1**	65.6	40.3	(75.2)	(49.9)	(61.8)	(55.1)	**77.7**	61.0	81.6	**69.8**	**81.5**	67.4	78.2	**72.4**	**85.1**	76.8	**84.8**	**80.6**
edit	AIMed	(75.2)	(68.8)	(27.7)	(39.0)	67.5	**86.4**	28.8	15.9	**78.1**	**87.0**	24.5	38.3	71.1	**92.9**	23.9	27.5	73.2	75.0	21.8	3.6
	BioInfer	66.9	**30.0**	58.4	39.6	(67.4)	(50.4)	(39.2)	(43.8)	72.7	59.4	65.6	62.4	69.3	61.1	55.8	58.4	66.9	69.0	54.3	60.8
APG	AIMed	(84.6)	(59.9)	(53.6)	(56.2)	66.0	56.5	14.0	22.5	**77.7**	74.1	52.8	61.6	73.1	69.2	13.4	22.5	82.7	**88.9**	29.8	17.6
	BioInfer	71.2	24.7	81.8	37.9	(81.5)	(60.2)	(61.3)	(60.7)	76.0	49.3	**84.0**	62.1	**81.4**	61.7	**82.7**	70.7	82.0	69.0	**85.4**	76.3

CC results trained on the 3 smaller corpora are shown in the Supplement, Table S5. Classifiers are trained on one corpus and tested on the other four corpora. Rows correspond to the training corpora and columns to test corpora. For reference, cross-validated results are shown in parentheses. Bold typeface highlights overall best results per corpus (differences under 1 base point are ignored).

Overall, our expectation that average CC performance would be worse than CL performance because of the smaller size of training data was in general not confirmed. On the one hand, the average performance measured across all four possible training corpora drops for SL, kBSPS, and APG kernels (the magnitude of the drop increases in this order), while it increases for SpT and edit, so the difference between the performance of these groups shrinks. On the other hand, the average CC F-score belonging to the best training corpus is somewhat better than the average CL F-score also for SL, kBSPS and APG, while AUC decreases slightly.

**Figure 9:**
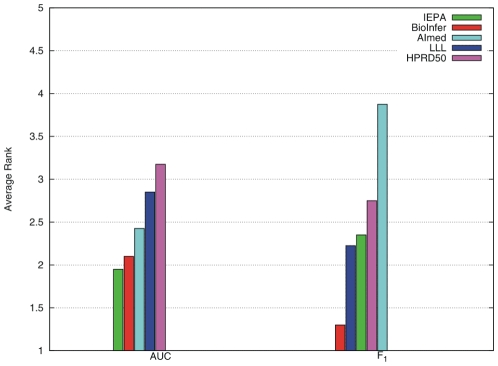
Overall ranking of the 5 corpora from the generality perspective in terms of the main performance measures based on the CL evaluation. The ranking are calculated as the average of rankings on the 5 selected kernels (see Table S6).

The CC results show large performance differences for most kernels depending on the training corpus. From cross-corpus evaluation, we can estimate which corpora is the best resource from a generalization perspective. We rank each training corpus for each kernel and average these numbers to obtain an overall rank (Table S6, Figure 9). This ranking only roughly reflects the size of the corpora. BioInfer, containing the most PPI pairs, gives the best performance with most kernels, and its overall rank calculated over the five kernels is 2.1 (AUC) and 1.3 (F). Surprisingly, systems trained on IEPA perform on average quite well, though IEPA is an order of magnitude smaller than AIMed or BioInfer. In contrast, AIMed is, despite its size, only the third best corpus in terms of AUC and by far the worst for F-score. This does not mean that AIMed is a bad choice for training, but only that differs from the other corpora: the ratio of positive/negative examples is the smallest, and it has the largest fraction of sentences with no interactions.

## Discussion

We performed a systematic benchmark of nine different methods for the extraction of protein-protein-interactions from text using five different evaluation corpora. All figures we report were produced using locally installed, trained, and tuned systems (the packages are available in the online appendix). In almost all cases, our results in cross-validation are well in-line with those published in the respective original papers; only in some cases we observed differences larger than 2%, and those could be attributed to different evaluation methods and different tuning procedures (see Results, Cross-validation). In contrast to cross-validation, our results regarding cross-learning and cross-corpus settings mostly cannot be compared to those of others as such numbers do not exist.

### Relative Performance of Kernels

Taking all our results into account (summarized in Table 5), we can safely state that APG, SL and kBSPS kernels are superior to the other methods we tested. APG provides on average the best AUC scores over all experiments when trained with the AUC-optimized sparse RLS. Its AUC scores drop significantly when trained with SVM. There is only one experiment where APG-RLS is outperformed by another method by a clear margin (CL on BioInfer). SL and kBSPS are on par in CL evaluation, while SL is slightly more accurate at CV. The ranking of kernels based on F-score is more diverse. At the more important CL evaluation, the clear advantage of APG observed at CV vanishes against kBSPS. Similarly, SL produces significantly better F-score at CV than at CL evaluation. Only these top-3 performing kernels outperform the rule-based RelEx approach (recall that RelEx's classification model is corpus-independent and thus can be used as baseline in all evaluation settings), at least in CV evaluation. When the more realistic CL evaluation is used, the best methods only just reach or marginally overcome RelEx's accuracy.

**Table 5:** Comparison of CV, CL, and CC results of selected kernels.

	AIMed		BioInfer	
Kernel	AUC	F	AUC	F
	CV/CL/CC	CV/CL/CC	CV/CL/CC	CV/CL/CC
SL	83.5/**77.5**/**76.8**	54.5/42.6/**41.5**	**81.1**/**74.9**/**73.1**	**60.0**/46.2/**40.6**
SpT	66.1/56.8/65.3	27.3/28.6/34.7	74.1/64.2/69.5	53.4/43.0/24.3
kBSPS	75.1/72.1/71.8	44.6/40.3/40.3	75.2/73.3/69.9	55.1/**47.6**/24.8
edit	75.2/62.8/66.9	39.0/37.0/39.6	67.4/61.0/67.5	43.8/31.7/15.9
APG	**84.6**/**77.6**/71.2	**56.2**/**43.8**/37.9	**81.5**/69.6/66.0	**60.7**/39.1/22.5

CC results for AIMed (resp. BioInfer) are obtained with classifier trained on BioInfer (resp. AIMed).

The performance of the other six kernels is clearly weaker. Kernels using syntax trees are on par with simple co-occurrence for CV, and their performance decreases drastically at CL evaluation. Cosine and edit kernels are slightly better than co-occurrence in CV, but their performance also drops significantly in CL evaluation.

**Figure 10:**
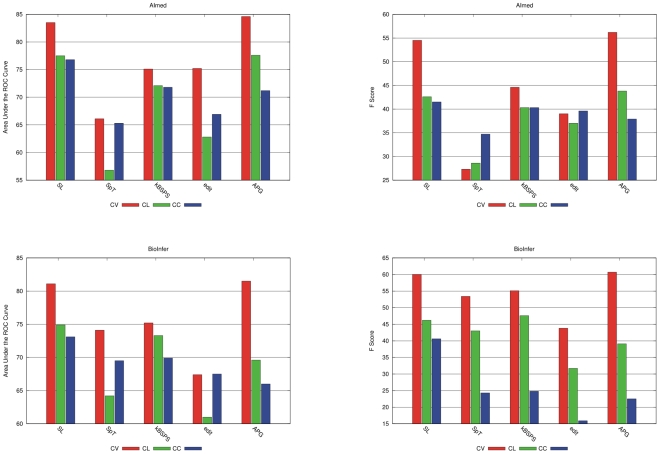
Performance comparison of SL, SpT, kBSPS, edit and APG kernels across CV, CL and CC evaluations. AUC and F-score values on AIMed and BioInfer. CC values are obtained with training on the other large corpus, though, eventually training on a smaller corpora may yield better results.

Table 5 clearly shows that performance drops considerably for all kernels from CV to CL (see also Figure 10). There also is strong tendency to a worse performance when switching from CL to CC in terms of F-score, but this tendency has many exceptions for AUC. The general decrease in performance can be attributed both to overlearning on the training set (in other words missing generalization capability) and to the significant differences among corpus characteristics. The magnitude of the decrease varies by kernels (CV to CL): From the top-3 kernels, kBSPS has the least decline, while SL shows the largest drop. APG has particularly low scores on the BioInfer corpus compared to other experiments. This corpus exhibits the average largest drop from CV to CL, which can be explained by the fact that it has the largest number of PPI pairs and that a remarkable portion of those pairs is uncovered by the patterns of other corpora.

### Diversity of Corpora

The performance of machine-learning methods to PPI extraction largely depends on the specific relationship between the training data and the data the method is used on later (test data). If these two data sets exhibit large differences, then evaluation results obtained using only the training data will be much different than those obtained when using the trained model on the test data. Differences can be, among other, the style of writing, the frequency of certain linguistic phenomena, or the level of technical detail in the texts. For the case of PPI, important differences are the ratio between sentences containing a PPI and those that do not, or the implicit understanding of what actually is a PPI—this might, for instance, include or exclude temporary interactions, protein transport, functional association only hinting, yet not proving a physical contact etc; see [36, Table 1] for more details.

Our experiments in CL and CC setting show, in accordance with results obtained by others [36], that the five corpora used for evaluation indeed have different characteristics. The main source of differences stems from the different ratio of positive pairs to negative pairs. The AIMed corpus has the largest fraction of negative sentences; accordingly, models trained on this corpus are more conservative in predicting PPIs, which leads to a lower recall when those models are applied on corpora with a smaller fraction of negative sentences. Both CL and CC evaluation clearly confirm this behavior, giving the AIMed corpus a bit of an outsider status. To further test this hypothesis, we repeated the CL experiment discarding AIMed from the learning pool. This leads to a significant increase in average F-measures (see details in Supplement; Text S2 and Table S7), confirming the special role of AIMed.

However, also the other corpora are not homogeneous. This becomes especially clear when comparing CV results with those from CL and CC evaluations. As explained before, in CV all characteristics of the test corpus are also present in the training corpus and are thus learned by the algorithms; in contrast, in CL and CC this is not the case. The relatively large differences in the obtained performance measures indicate that different corpora have notably different characteristics. As any new texts that PPI extraction algorithms would be applied on would have unknown characteristics, we conclude that only the performance we measured for CL and CC can be expected on such texts.

### Shallow Parsing versus Syntax Trees versus Dependency Graphs

We evaluated kernels based on shallow linguistic features, syntax tree, dependency graph, and mixtures of these three. Our results clearly show that syntax trees are less useful than the other representations. Recall that syntax trees contain no explicit information about the semantic relations of connected nodes, which apparently is crucial in a relation extraction task. For the other types of data, the picture is less clear.

Several authors claimed that using more types of information yields better performance [37], [63]. Our experiments only partially confirm this claim. In cross-validation results, the APG kernel, which combines multiple sources of sentence information, shows the best performance among all analyzed kernels in terms of both AUC (only with RLS) and F-score. However, this advantage shrinks (AUC) or vanishes (F-score) for cross-corpus and cross-learning evaluation when compared to the pure dependency graph-based kBSPS. We conclude that using more information in first place helps in becoming more corpus-specific. However, this situation might be different if larger training corpora were available.

In contrast to APG and kBSPS kernel, the SL kernel does not use any deep parse information. Nevertheless, it produces results comparable with APG and better than kBSPS for cross-validation. Its superiority over kBSPS vanishes for cross-learning, however. This change may be attributed to the decreasing usefulness of shallow linguistic features—including word sequences—when the model is trained on a more heterogeneous corpus.

Our results also show that the descriptive power of dependency graph parses can only be exploited when combined with an appropriate kernel. Cosine and edit kernels are unable to efficiently capture the features from dependency graphs. In case of the former, the shortcoming may be accounted to the fact that cosine does not take the word order into account. The handicap of the latter can be explained by weighting scheme applied at path distance calculation: its uniform, grammar-independent weighting disregards grammatical rules and structures, and thus the semantics of the underlying text.

### AUC or F-Measure?

Recently, some authors criticized the F-score as performance measure, because it is very sensitive to the ratio of positive/negative pairs in the corpus [17], , and it is less stable to parameter modifications than AUC. Our experiments confirm both statements. The standard deviation of AUC in CV across the five corpora ranges between 1.34 and 7.45 (F-score: 8.36–24.04). The SL and APG kernels are the most stable ones, while SpT and edit kernels belong to the other extreme in terms of both measures. Figure 6 depicts the robustness on corpus level for cross-validation. We can observe that the larger the corpus the smaller the standard deviation, independently from the applied kernel.

On the other hand, one must keep in mind that AUC is a statement about the general capabilities of a PPI extraction method that must not be confused with its expected performance on a concrete problem. For a concrete task, a concrete set of parameters has to be chosen, while AUC expresses a measure over a range of parameter settings. When a user wants to analyze a set of documents, one probably can safely advise her to prefer kernels with higher average AUC measure, but the achieved performance will depend very much on the concrete parameters chosen. We also show via the APG-SVM experiment that the AUC score depends very much on the learning algorithm of the classifier, and only partially on the kernel. Therefore, the (less stable) F-score actually gives a better picture on the expected performance on new texts.

### Robustness against Parameter Setting

We investigated the robustness of the different kernels against parameter settings. To this end, we performed exhaustive, fine-grained parameter optimization for selected tasks and measured the difference to the parameter setting used in the benchmark. The resulting picture is quite heterogeneous.

SL kernel in principle has a number of parameters, but the implementation we were provided with from the authors always uses a default setting (which yields sound results). Therefore, we could not test robustness of SL in terms of parameter settings.

When using task-specific parameter tuning at CV for syntax-tree-based kernels, an improvement of 3 (5) points can be achieved on AUC (F-score). The magnitude of improvement is larger on CL, but the figures remain low. On the other hand, with improper parameter setting, the F-score may drop drastically, even to 0. Overall, syntax-tree-based kernels behave very sensitive to parameter setting.

A fine-grained parameter tuning improves kBSPS results only insignificantly (1–3 points of improvement both AUC and F). A similar small drop can be observed at CV evaluation if the parameters are selected improperly, while at CL evaluation the drop gets larger and reaches 10–15 points F-score. Consequently, we can state that kBSPS is fairly robust to parameter selection.

Cosine and edit show significantly better (high 60s/low 70s) AUC values with task-specific parameter tuning at CL evaluation, but those settings cause a dramatic F-score decrease (cosine: 20–25, edit 6–12 points). At CV evaluation, the trend is similar, but the extent of changes is smaller. As a summary, cosine and edit also should be considered as sensitive to parameter settings.

The performance of APG hardly changes (1–2 points) if the parameters are set differently (CV). The F-score drop is somewhat larger at CL. On the other hand, a major F-score drop can be observed when the threshold parameter is not optimized. When trained with SVM, APG becomes even more sensitive to the right selection of parameters.

### Classification Time

The runtime of a kernel-based method has two main components. First, the linguistic structures have to be generated. Previous experiments show [37, Table 2] that dependency parsers can be about an order of magnitude faster than syntax parsers and shallow parsing is about 1.5 order of magnitude faster than dependency parsing (see [65, Table 2]). Second, the substructures used by the kernels have to be determined and the classifier has to be applied.

We give an overview of the theoretical complexity of each kernel in the Supporting Information (Text S3). Actual runtimes are probably more interesting, as the complexity of an algorithm can be distorted to a large degree by the quality of its implementation. We show in Table S9 averaged training and test times for each corpus for CV settings. Note that these figures do not contain the time it takes to parse a sentence; thus, real runtimes would be much higher for all kernels except SL. The APG with its cubic complexity clearly has the longest training time, but the classifier is fast. PT kernel generates the most syntax tree substructures and is an order of magnitude slower both in terms of training and classification time. We can also see that kernels with linear complexity exhibit very different runtimes. Among them kBSPS clearly is the fastest both at training and classification.

Runtime is a strong argument when it comes to the application of a PPI extraction method on large corpora. Consider the top-3 kernels AGP, SL, and kBSPS. When applied to all of Medline with its approximately 120M sentences, one would expect runtimes of 45, 141, and 4 days, respectively, on a single processor and I/O stream. Taking also into account the computation of shallow parses and dependency trees (on average 4 ms and 130 ms per sentence, respectively), times change to 226, 147, and 185 days, thus the formerly existing large differences almost vanish. Clearly, the exact times depend on the hardware that is used, but the ratios should stay roughly the same. The figures imply that an application of kernel based methods to PPI extraction on large corpora is only possible if considerable computational resources are available.

### Summary Kernel-by-Kernel

The SL kernel uses only shallow linguistic information plus the usual bag-of-word features. Taking parse time into account, this kernel is the fastest among all we tested. Despite its simplicity, its performance is remarkable. It is on par with the best kernels in most of the evaluation settings, and yields particularly good results in CV — all with default parameter settings. Furthermore, its performance is the most robust on the two larger corpora across CV, CL and CC evaluation in terms of both AUC and F-score.

Syntax-tree-based kernels (ST, SST, PT, SpT) fail to achieve comparative performance. Their performance hardly reaches the baseline even at CV evaluation. They are also very sensitive to the parameter setting and have a long runtime. Results are very sensitive to the particular training/test corpora and therefore cannot be extrapolated safely to new texts.

The kBSPS kernel achieves an overall very good performance, particularly in the more important CL and CC evaluations. Its performance decreases the least when CL evaluation is used instead of CV. It is very robust against parameter settings and achieves very good results with default parameters. Furthermore, it is by far the fastest kernel among all that use rich linguistic information.

Cosine and edit kernels, though using dependency trees, show significantly worse performance than the top-3 kernels. They are also very sensitive to the parameter settings. Their runtime is the double compared to other dependency tree kernels. In [22] Erkan proposed to train these kernels with transductive SVM [66], however the performance gain is dubious (see Table S8), while increases tremendously the training time.

APG shows the best performance at CV setting, but its superiority vanishes on the more important CL and CC settings. It uses a different learner than other kernels, which optimizes for AUC. Consequently, its AUC results are the best, but its F-score values are also good (CV, and partly CL). Recall when APG is trained with SVM its AUC performance drops significantly compared to APG-RLS. This reflects the fact that RLS specifically optimizes for AUC; in turn, one can expect other kernels to also obtain better results when RLS learning would be applied. APG is rather sensitive to evaluation settings, where is exhibits the largest drop among top-3 kernels. It is robust to parameter settings except the threshold for the RLS procedure, but becomes very sensitive to parameters when trained with SVM. The classification is pretty fast, but with the necessary preprocessing, it becomes the slowest of the top-3 kernels.

### Conclusion

We investigated nine kernel-based methods for the extraction of PPIs from scientific texts. We studied how these methods behave in different evaluation settings, using different parameter sets, and on different gold standard corpora. We showed that even the best performing kernels, requiring extensive parameter optimizations and large training corpora, cannot be considered as significantly better than a simple rule-based method which does not need any training at all and has essentially no parameters to tune. We also showed that the characteristic features of PPIs can be extracted much more efficiently by kernels based on dependency tree parses than by those based on syntax tree parses. Interestingly, the SL kernel, using only shallow linguistic analysis, is almost as good as the best dependency-based kernels. We pointed out that the advantage of APG kernel, using multiple representations as features, vanishes in a realistic evaluation scenario when compared to the simpler kBSPS and SL kernels.

The ultimate goal of this study was to select the best PPI extraction method for real applications and to generate performance estimates for this method (and others) on new text. We state that this goal was not achieved for mostly two reasons. First, the performance of the methods we studied is very sensitive to parameter settings, evaluation method, and evaluation corpus. Best scores are only achieved when settings are optimized against a gold standard kernel—something that is not possible on unseen text. Our results reveal that some methods apparently are better than others, but a clear-cut winner is not detectable given the bandwidth of results. Second, the heterogeneity between corpora leads to extremely heterogeneous evaluation results, showing that all methods strongly adapt to the training set, and that, in turn, the existing training corpora are not large or not general enough to capture the characteristics of the respective other corpora. This implies that any extrapolation of the observed scores (AUC or F-score) to unseen texts is questionable.

We believe that these findings call for a number of actions. First, there is a strong need to create larger and better characterized evaluation corpora. Second, we think that there is also a need to complement the currently predominant approach, treating all interactions as equally important, with more specific extraction tasks. To this end, it is important to create specialized corpora, such as those for the extraction of regulation events or for protein complex formation. The more specific a question is, the simpler it is to create representative corpora, leading to better models, often higher extraction performance and better comparability of methods. For instance, works like [67] on extraction of gene regulation or [68] on extraction of phosphorylation events report much higher accuracies than those current achievable in the general PPI task. Third, there is a severe lack in studies measuring real-life performance of PPI extraction methods, circumventing the usage of gold standards by, for instance, user surveys with biological experts. Last but not least, our result also show that rule-based methods still make an excellent stand when compared to machine-learning based approaches as soon as specific evaluation settings are left behind.

## Supporting Information

Table S1 Overview of the evaluated kernels. Overview of the nine kernels evaluated in the paper.
(0.07 MB PDF)

Table S2 Other kernels considered. Overview of other kernel based methods in the literature that we did not tested in the paper.
(0.06 MB PDF)

Table S3 Overview of the usability of the different kernels. Some details on the nine evaluated kernels: availability of the algorithm and documentation, type of learning software.
(0.06 MB PDF)

Table S4 Overview of our parameter selection strategy. Overview of our parameter selection strategy used in the paper. We provide a coarse description of parameter ranges and best parameters for each kernel and evaluation setting.
(0.07 MB PDF)

Table S5 Cross-corpus results. Full table of cross-corpus results trained on all 5 corpora and evaluated on all nine kernels.
(0.08 MB PDF)

Table S6 Ranking of corpora at CC evaluation based on their AUC and F-score values. We ranked the corpora from the generality perspective, i.e. how general the systems are trained on specific corpora. The evaluation is based on their AUC and F-score values at CC evaluation.
(0.07 MB PDF)

Table S7 Cross-learning experiments with some selected kernel performed on 4 corpora (all but AIMed). Cross-learning experiments with some selected kernel performed on 4 corpora (all but AIMed). Classifiers are trained on the ensemble of three corpora and tested on the forth one.
(0.07 MB PDF)

Table S8 CV results with transductive SVM for kBSPS, edit, cosine kernels. Results with the transductive learning strategy for some selected kernels.
(0.06 MB PDF)

Table S9 Average runtime of training and test processes, and runtime estimates on entire Medline. Average runtime of training and test processes per corpus measured over all cross-validation experiments for each kernel (not including the parsing time at pre-processing), and rough runtime estimates on the entire Medline.
(0.06 MB PDF)

Text S1 Similarity function in kBSPS kernel. Definition of similarity score used in kBSPS kernel.
(0.07 MB PDF)

Text S2 Additional experiments. We provide here details of two additional experiments. (1) Cross-learning (CL) without AIMed, that is systems are trained on 3 corpora and tested on the fourth one. (2) Models trained with transductive SVM.
(0.05 MB PDF)

Text S3 Theoretical complexity of kernels. We provide here details on the computational complexity of kernels.
(0.04 MB PDF)
