# Supplementary material for: A Comprehensive Benchmark of Kernel Methods to Extract Protein–Protein Interactions from Literature
Source: PLoS Comput Biol. 2010 Jul 1;6(7):e1000837. doi: 10.1371/journal.pcbi.1000837 (PMC2895635; doi:10.1371/journal.pcbi.1000837)
Supplement: Table S1 — Overview of the evaluated kernels. Overview of the nine kernels evaluated in the paper. (0.07 MB PDF) [file pcbi.1000837.s001.pdf]

Table S1. Overview of the evaluated kernels

Kernel	Reference	Abbreviation	Information source	Features
shallow linguistic	[23]	SL	lexical, shallow	fore-between, between and between-after tokens relative to the entity pair; POS, lemma, orthographic features of tokens in the w size window of the entities
subtree	[30]	ST	syntax	subtrees of the syntax tree (see Figure 1)
subset tree	[20]	SST	syntax	subset trees of the syntax tree (see Figure 1)
partial tree	[28]	PT	syntax	partial trees of the syntax tree (see Figure 1)
spectrum tree	[58]	SpT	syntax	directed vertex-walks of length q (q-grams) of the syntax tree (see Figure 1)
k-band shortest path spectrum	[29]	kBSPS	dependency	vertex-walks of size in the range $[q_{\min}, q_{\max}]$ along the k-band shortest path between the entity pair in the dependency tree
cosine similarity	[22]	cosine	dependency	shortest path between the entity pair of the dependency tree
edit distance	[22]	edit	dependency	shortest path between the entity pair of the dependency tree
all-paths graph	[17]	APG	shallow, dependency	full dependency graph with POS tags, full word sequence as graph with POS tags, all edges are weighted, higher weights along the shortest path in the dependency graph

Legend: shallow – shallow linguistic information, such as lemma, stem, POS-tag, usually in conjunction with bag-of-words or token sequence representation; syntax – syntax tree parses; dependency – dependency tree or graph parses; lexical – gazetteers of clue words, e.g. for interactions.
