# Supplementary material for: A Comprehensive Benchmark of Kernel Methods to Extract Protein–Protein Interactions from Literature
Source: PLoS Comput Biol. 2010 Jul 1;6(7):e1000837. doi: 10.1371/journal.pcbi.1000837 (PMC2895635; doi:10.1371/journal.pcbi.1000837)
Supplement: Table S2 — Other kernels considered. Overview of other kernel based methods in the literature that we did not tested in the paper. (0.06 MB PDF) [file pcbi.1000837.s002.pdf]

Table S2. Other kernels considered

Kernel(s)	Reference	Information source	Reason why not evaluated
predicate, walk, dependency, hybrid kernels	[25]	shallow, lexical, dependency	Not available. The link to the source is broken and the authors did not reply to our enquiries.
rich-feature-vector-based kernel	[26]	shallow, dependency	Not available. The source code will be published later as a part of a package (S. van Landeghem, personal communication).
kernel based on even more features	[67]	lexical, dependency	Only the feature selection is published at `http://www.cs.utoronto.ca/~juris/data/BI09/`
subtree on dependency trees	[21]	dependency	Not available.
contiguous and sparse subtree kernels	[32]	dependency	Not available.
general sparse subsequence kernel	[19]	shallow	Similar to [23], but shows lower performance.
convolution dependency path kernel	[31]	dependency	Not available.
hybrid kernel	[27]	shallow, syntax, dependency	Not available. The authors plan to publish their approach soon (M. Miwa, personal communication) `http://www-tsujii.is.s.u-tokyo.ac.jp/~satre/akane/`
local alignment kernel	[24]	shallow	Not available. May be published in the future (S. Katrenko, pers. communication)

For the description of information sources, see Table S1.
