# Supplementary material for: A Comprehensive Benchmark of Kernel Methods to Extract Protein–Protein Interactions from Literature
Source: PLoS Comput Biol. 2010 Jul 1;6(7):e1000837. doi: 10.1371/journal.pcbi.1000837 (PMC2895635; doi:10.1371/journal.pcbi.1000837)
Supplement: Table S3 — Overview of the usability of the different kernels. Some details on the nine evaluated kernels: availability of the algorithm and documentation, type of learning software. (0.06 MB PDF) [file pcbi.1000837.s003.pdf]

Table S3. Overview of the usability of the different kernels

Name	Availability	Learning software	Documentation
SL	http://tcc.itc.it/research/textec/tools-resources/jsre/user-guide-1.1.html	libSVM 2.80	homepage and readme
ST	http://dit.unitn.it/~moschitt/Tree-Kernel.htm	SVM-lighT-TK 1.5	homepage
SST	http://dit.unitn.it/~moschitt/Tree-Kernel.htm	SVM-lighT-TK 1.5	homepage
PT	on request	SVM-lighT-TK 1.5	homepage
SpT	published in the online appendix of this paper; implemented by [29]	SVM-Light 6.02	homepage
kBSPS	published in the online appendix of this paper; implemented by [29]	SVM-Light 6.02	homepage
cosine	on request	SVM-Light 6.01	readme file
edit	on request	SVM-Light 6.01	readme file
APG	http://mars.cs.utu.fi/PPICorpora/GraphKernel.html	sparse RLS	readme file

All tools are command-line-based. Solely APG uses the uniform XML training format from [37], therefore for all other tools the input files have to be appropriately converted.
