# Supplementary material for: A Comprehensive Benchmark of Kernel Methods to Extract Protein–Protein Interactions from Literature
Source: PLoS Comput Biol. 2010 Jul 1;6(7):e1000837. doi: 10.1371/journal.pcbi.1000837 (PMC2895635; doi:10.1371/journal.pcbi.1000837)
Supplement: Table S4 — Overview of our parameter selection strategy. Overview of our parameter selection strategy used in the paper. We provide a coarse description of parameter ranges and best parameters for each kernel and evaluation setting. (0.07 MB PDF) [file pcbi.1000837.s004.pdf]

Table S4. Overview of our parameter selection strategy

Kernel	Parameters	Range	Default	Average best	Remark
SL					the implementation disregards the parameter setting, always runs with the default
ST<br>SST<br>PT	c; j; λ; μ	[1,16,64,128]; [0.5,1,2]; [0.2,0.4,0.8]; [0.2,0.4]	$0; 1; 0.4; 0.4$	$1; 2; 0.4; 0.4$<br>$1; 2; 0.4; 0.4$<br>$16; 2; 0.4; 0.4$	the default $c = 0$ yields often 0 F-score. In [29] the experiments with $c = 2^i$, $i = -6, \ldots, -1$ yielded inferior results
SpT	c; j; q	$2^i, i = -6, \ldots, 6$; [0.5,1,2]; [2]	*; $1; 10$	CV: default; $2; 2$<br>CL: $64; 1; 2$	*: the default c is the reciprocal of the average norm of training examples; q is the length of v-walks, its default is not reasonable for PPI extraction
kBSPS	L; E; D; l; e; d; $q_{\min}$; $q_{\max}$; k; j	[0]; [0]; [0,−1]; [1,3,6]; [1,3,6]; [1,3,6]; [1,2]; [2,3]; [0,1]; [1,2]	0; 0; −1; 1; 6; 6 2; 2; 0; 2	CV: D=0, d=3, all others default;<br>CC, CL: default	c was set to the default as for SpT
cosine<br>edit	c	[0,1,10,100]	0	CV: 10<br>CC, CL: 100	Due to the modest results obtained, we did not perform more comprehensive optimization
APG	c; svnum; token; vector	[0.25;0.5;1;2]; [500,2000]; [split,Charniak]; [linearized, normalized]		CV/CL/CC: 0.25/1/1; 2000/2000/2000; s/c/s; n/n/l	APG comes with no default;<br>svnum = number of support vectors<br>for threshold specification we used the script provided with the implementation
APG (SVM)	c; j	[2^i, $i = -10, \ldots, 10$]; [1,2]		CV: 1;2	APG trained with SVMlight, other parameters as with standard APG
