# Supplementary material for: A Comprehensive Benchmark of Kernel Methods to Extract Protein–Protein Interactions from Literature
Source: PLoS Comput Biol. 2010 Jul 1;6(7):e1000837. doi: 10.1371/journal.pcbi.1000837 (PMC2895635; doi:10.1371/journal.pcbi.1000837)
Supplement: Table S5 — Cross-corpus results. Full table of cross-corpus results trained on all 5 corpora and evaluated on all nine kernels. (0.08 MB PDF) [file pcbi.1000837.s005.pdf]

Table S5. Cross-corpus results

Kernel	Training corpus	AIMed				BioInfer				HPRD50				IEPA				LLL			
		AUC	P	R	F	AUC	P	R	F	AUC	P	R	F	AUC	P	R	F	AUC	P	R	F
SL	AIMed	(83.5)	(47.5)	(65.5)	(54.5)	**73.1**	66.8	29.2	40.6	72.9	61.7	56.4	59.0	68.8	66.3	15.8	25.5	72.6	86.4	23.2	36.5
	BioInfer	**76.8**	27.2	87.1	**41.5**	(81.1)	(55.1)	(66.5)	(60.0)	74.8	51.0	78.5	61.8	76.6	63.3	64.8	64.0	80.5	71.5	78.0	74.6
	HPRD50	69.4	29.8	53.2	38.2	67.9	47.7	34.3	39.9	(80.0)	(64.4)	(67.0)	(64.2)	69.5	64.0	45.7	53.3	74.2	70.6	54.3	61.4
	IEPA	65.8	25.9	46.0	33.1	**72.7**	55.0	36.9	44.2	71.4	60.1	56.4	58.2	(81.1)	(69.5)	(71.2)	(69.3)	76.3	80.2	56.7	66.4
	LLL	61.8	21.4	66.6	32.4	68.1	40.5	58.8	47.9	72.0	50.4	71.2	59.0	72.5	52.8	77.0	62.6	(81.2)	(69.0)	(85.3)	(74.5)
ST	AIMed	(68.9)	(40.3)	(25.5)	(30.9)	60.8	49.0	13.7	21.4	59.9	45.0	46.6	45.8	61.8	69.4	22.4	33.9	48.0	78.6	6.7	12.4
	BioInfer	63.7	22.5	65.8	33.5	(74.2)	(46.8)	(60.0)	(52.2)	55.3	41.9	23.9	30.5	52.5	51.5	15.2	23.5	58.0	51.2	67.1	58.1
	HPRD50	59.7	23.0	49.3	31.4	59.4	40.8	36.5	38.5	(63.3)	(49.7)	(67.8)	(54.5)	64.5	57.8	48.7	52.8	48.9	49.3	40.9	44.7
	IEPA	62.3	29.8	23.0	26.0	65.3	54.0	15.2	23.7	56.2	53.4	33.7	41.4	(75.8)	(59.4)	(75.6)	(65.9)	68.6	73.5	30.5	43.1
	LLL	60.4	23.3	55.9	32.9	64.3	38.7	38.1	38.4	58.3	43.0	71.8	53.8	69.0	47.0	89.6	61.7	(69.0)	(55.9)	(100)	(70.3)
SST	AIMed	(68.9)	(42.6)	(19.4)	(26.2)	65.6	67.3	7.4	13.3	60.1	56.8	15.3	24.2	60.6	50.0	0.6	1.2	60.9	90.9	6.1	11.4
	BioInfer	66.0	22.0	76.5	34.1	(73.6)	(47.0)	(54.3)	(50.1)	60.9	46.0	31.3	37.2	59.2	56.9	27.2	36.8	67.8	60.1	70.7	65.0
	HPRD50	59.9	22.2	54.9	31.6	55.2	40.1	36.2	38.0	(62.2)	(48.1)	(63.8)	(52.2)	65.8	52.6	62.7	57.2	56.2	58.3	42.7	49.3
	IEPA	63.3	27.7	39.8	32.7	60.8	47.2	21.9	29.9	63.3	47.1	60.7	53.1	(72.4)	(54.8)	(76.9)	(63.4)	73.4	74.3	45.7	56.6
	LLL	60.9	17.1	**100.**	29.3	65.1	26.2	**100.**	41.5	58.5	37.6	**100.**	54.7	65.6	41.0	**100.**	58.2	(63.8)	(55.9)	(100)	(70.3)
PT	AIMed	(68.5)	(39.2)	(31.9)	(34.6)	58.4	47.8	10.9	17.8	66.2	56.2	41.7	47.9	66.1	67.5	15.5	25.2	55.2	**92.3**	7.3	13.6
	BioInfer	65.9	23.0	69.5	34.6	(73.8)	(45.3)	(58.1)	(50.5)	59.8	44.1	34.4	38.6	61.5	58.5	16.4	25.6	59.1	52.8	70.1	60.2
	HPRD50	64.7	29.4	45.6	35.8	61.9	43.6	22.5	29.7	(65.2)	(54.9)	(56.7)	(52.4)	69.0	60.1	54.3	57.1	54.7	51.9	33.5	40.7
	IEPA	63.8	31.6	25.8	28.4	65.6	50.8	12.3	19.8	62.3	50.4	37.4	43.0	(73.1)	(63.1)	(66.3)	(63.8)	69.8	75.8	30.5	43.5
	LLL	59.4	18.6	76.6	30.0	63.0	32.1	66.8	43.3	59.9	43.1	77.9	55.5	59.0	42.8	77.6	55.2	(66.7)	(56.2)	(97.3)	(69.3)
SpT	AIMed	(66.1)	(33.0)	(25.5)	(27.3)	69.5	48.4	16.3	24.3	60.0	47.1	39.9	43.2	67.9	59.7	11.0	18.6	57.0	72.7	9.8	17.2
	BioInfer	65.3	22.3	77.8	34.7	(74.1)	(44.0)	(68.2)	(53.4)	57.2	41.4	67.5	51.3	69.9	61.2	52.2	56.4	55.7	54.2	62.8	58.2
	HPRD50	62.0	22.6	57.8	32.5	65.1	39.7	40.4	40.0	(65.7)	(49.3)	(71.7)	(56.4)	69.2	57.8	53.1	55.4	57.0	58.3	42.7	49.3
	IEPA	64.2	28.5	38.3	32.7	72.1	52.6	22.1	31.1	60.7	45.3	53.4	49.0	(75.9)	(54.5)	(81.8)	(64.7)	72.1	73.2	43.3	54.4
	LLL	64.5	19.2	90.7	31.7	70.9	35.1	88.4	**50.3**	57.3	37.6	100.	54.7	72.7	42.3	97.3	59.0	(50.0)	(55.9)	(100)	(70.3)
kBSPS	AIMed	(75.1)	(50.1)	(41.4)	(44.6)	69.9	71.6	15.0	24.8	76.8	77.5	38.0	51.0	73.6	66.7	5.4	9.9	75.1	85.7	7.3	13.5
	BioInfer	71.8	29.1	65.6	40.3	(75.2)	(49.9)	(61.8)	(55.1)	**77.7**	61.0	81.6	**69.8**	**81.5**	67.4	78.2	**72.4**	85.1	76.8	84.8	**80.6**
	HPRD50	66.4	24.9	64.1	35.9	66.1	38.3	53.6	44.7	(79.3)	(62.2)	(87.1)	(71.0)	69.0	55.3	56.1	55.7	71.2	65.1	59.2	62.0
	IEPA	67.0	23.1	77.2	35.6	71.4	40.1	70.7	**51.1**	74.9	52.1	84.1	64.3	(83.2)	(58.8)	(89.7)	(70.5)	80.7	64.5	**92.1**	75.9
	LLL	60.1	22.4	75.3	34.5	67.7	37.0	71.8	48.8	72.3	49.8	84.1	62.6	74.6	53.0	89.6	66.6	(84.3)	(69.3)	(93.2)	(78.1)
cosine	AIMed	(70.5)	(43.6)	(39.4)	(40.9)	61.1	45.2	22.8	30.3	68.1	71.4	39.9	51.2	51.2	50.0	14.9	23.0	56.9	77.3	20.7	32.7
	BioInfer	65.4	26.3	59.7	36.5	(66.1)	(44.8)	(44.0)	(44.1)	70.8	56.6	71.2	63.0	57.1	47.9	40.0	43.6	67.3	71.3	59.2	64.7
	HPRD50	63.2	24.4	49.8	32.7	60.2	37.9	37.4	37.7	(74.8)	(59.0)	(67.2)	(61.2)	55.8	46.2	44.8	45.5	63.3	75.3	40.9	53.0
	IEPA	57.2	20.6	55.8	30.1	61.5	34.5	51.9	41.4	59.9	44.4	67.5	53.5	(75.5)	(61.3)	(68.4)	(64.1)	66.4	60.9	73.2	66.5
	LLL	57.1	19.9	63.2	30.2	61.4	33.4	58.8	42.6	66.6	45.9	71.8	56.0	70.6	53.8	78.5	63.8	(75.2)	(70.2)	(81.7)	(73.8)
edit	AIMed	(75.2)	(68.8)	(27.7)	(39.0)	67.5	**86.4**	8.8	15.9	**78.1**	**87.0**	24.5	38.3	71.1	**92.9**	3.9	7.5	73.2	75.0	1.8	3.6
	BioInfer	66.9	30.0	58.4	39.6	(67.4)	(50.4)	(39.2)	(43.8)	72.7	59.4	65.6	62.4	69.3	61.1	55.8	58.4	66.9	69.0	54.3	60.8
	HPRD50	68.0	**38.7**	40.1	39.4	65.4	57.4	21.9	31.7	(79.2)	(71.3)	(45.2)	(53.3)	70.9	79.8	28.4	41.9	77.9	89.8	32.3	47.5
	IEPA	66.4	32.4	35.8	34.0	67.9	60.8	28.2	38.5	75.6	67.3	42.9	52.4	(80.2)	(77.2)	(60.2)	(67.1)	**88.0**	90.1	50.0	64.3
	LLL	62.8	19.7	85.5	32.0	65.6	31.1	79.3	44.7	76.1	45.9	93.3	61.5	75.2	46.7	97.0	63.1	(87.5)	(68.0)	(98.0)	(78.4)
APG	AIMed	(84.6)	(59.9)	(53.6)	(56.2)	66.0	56.5	14.0	22.5	**77.7**	74.1	52.8	61.6	73.1	69.2	13.4	22.5	82.7	88.9	9.8	17.6
	BioInfer	71.2	24.7	81.8	37.9	(81.5)	(60.2)	(61.3)	(60.7)	76.0	49.3	84.0	62.1	81.4	61.7	82.7	70.7	82.0	69.0	85.4	76.3
	HPRD50	72.3	20.9	93.7	34.2	58.2	28.5	71.7	40.7	(80.9)	(68.2)	(69.8)	(67.8)	71.2	45.2	93.1	60.9	56.9	51.0	79.3	62.1
	IEPA	70.1	30.0	41.2	34.7	**72.7**	60.6	26.6	37.0	75.5	65.4	62.6	63.9	(83.9)	(66.6)	(82.6)	(73.1)	85.8	84.2	48.8	61.8
	LLL	61.1	21.9	59.2	32.0	65.7	35.2	65.5	45.8	73.5	54.8	74.2	63.0	72.9	53.7	76.1	63.0	(83.5)	(71.3)	(91.0)	(78.1)

Classifiers are trained on one corpus and tested on the other four corpora. Rows correspond to the training corpora and columns to test corpora. In parentheses, we show the cross-validated results for reference. Bold typeface indicates our best overall result for a corpus (differences under 1 base point are ignored).
