# Supplementary material for: A Comprehensive Benchmark of Kernel Methods to Extract Protein–Protein Interactions from Literature
Source: PLoS Comput Biol. 2010 Jul 1;6(7):e1000837. doi: 10.1371/journal.pcbi.1000837 (PMC2895635; doi:10.1371/journal.pcbi.1000837)
Supplement: Table S6 — Ranking of corpora at CC evaluation based on their AUC and F-score values. We ranked the corpora from the generality perspective, i.e. how general the systems are trained on specific corpora. The evaluation is based on their AUC and F-score values at CC evaluation. (0.07 MB PDF) [file pcbi.1000837.s006.pdf]

Table S6. Ranking of corpora at CC evaluation based on their AUC and F-score values

Kernel	Training corpus	AUC						F-score					
		AIMed	BioInfer	HPRD50	IEPA	LLL	rank	AIMed	BioInfer	HPRD50	IEPA	LLL	rank
SL	AIMed	–	**73.1** [1]	72.9 [2]	68.8 [4]	72.6 [4]	2.75	–	40.6 [3]	59.0 [2.5]	25.5 [4]	36.5 [4]	3.375
	**BioInfer**	**76.8** [1]	–	74.8 [1]	76.6 [1]	80.5 [1]	1	**41.5** [1]	–	61.8 [1]	64.0 [1]	74.6 [1]	1
	HPRD50	69.4 [2]	67.9 [4]	–	69.5 [3]	74.2 [3]	3	38.2 [2]	39.9 [4]	–	53.3 [3]	61.4 [3]	3
	IEPA	65.8 [3]	**72.7** [2]	71.4 [4]	–	76.3 [2]	2.75	33.1 [3]	44.2 [2]	58.2 [4]	–	66.4 [2]	2.75
	LLL	61.8 [4]	68.1 [3]	72.0 [3]	72.5 [2]	–	3	32.4 [4]	47.9 [1]	59.0 [2.5]	62.6 [2]	–	2.375
SpT	AIMed	–	69.5 [3]	60.0 [2]	67.9 [4]	57.0 [2.5]	2.875	–	24.3 [4]	43.2 [4]	18.6 [4]	17.2 [4]	4
	BioInfer	65.3 [1]	–	57.2 [4]	69.9 [2]	55.7 [4]	2.75	34.7 [1]	–	51.3 [2]	56.4 [2]	58.2 [1]	1.5
	HPRD50	62.0 [4]	65.1 [4]	–	69.2 [3]	57.0 [2.5]	3.375	32.5 [3]	40.0 [2]	–	55.4 [3]	49.3 [3]	2.75
	IEPA	64.2 [3]	72.1 [1]	60.7 [1]	–	72.1 [1]	1.5	32.7 [2]	31.1 [3]	49.0 [3]	–	54.4 [2]	2.5
	**LLL**	64.5 [2]	70.9 [2]	57.3 [3]	72.7 [1]	–	2	31.7 [4]	**50.3** [1]	54.7 [1]	59.0 [1]	–	1.75
kBSPS	AIMed	–	69.9 [2]	76.8 [3]	73.6 [3]	75.1 [3]	2.75	–	24.8 [4]	51.0 [4]	9.9 [4]	13.5 [4]	4
	**BioInfer**	71.8 [1]	–	**77.7** [2]	**81.5** [1]	85.1 [1]	1.25	40.3 [1]	–	**69.8** [1]	**72.4** [1]	**80.6** [1]	1
	HPRD50	66.4 [3]	66.1 [4]	–	69.0 [4]	71.2 [4]	3.75	35.9 [2]	44.7 [3]	–	55.7 [3]	62.0 [3]	2.75
	IEPA	67.0 [2]	71.0 [1]	**77.9** [1]	–	80.7 [2]	1.5	35.6 [3]	**51.1** [1]	64.3 [2]	–	75.9 [2]	2
	LLL	60.1 [4]	67.7 [3]	72.3 [4]	74.6 [2]	–	3.25	34.5 [4]	48.8 [2]	62.6 [3]	66.6 [2]	–	2.75
edit	AIMed	–	67.5 [2]	**78.1** [1]	71.1 [2]	73.2 [3]	2	–	15.9 [4]	38.3 [4]	7.5 [4]	3.6 [4]	4
	BioInfer	66.9 [2]	–	72.7 [4]	69.3 [4]	66.9 [4]	3.5	39.6 [1]	–	62.4 [1]	58.4 [2]	60.8 [2]	1.5
	HPRD50	68.0 [1]	65.4 [4]	–	70.9 [3]	77.9 [2]	2.5	39.4 [2]	31.7 [3]	–	41.9 [3]	47.5 [3]	2.75
	**IEPA**	66.4 [3]	67.9 [1]	75.6 [3]	–	**88.0** [1]	2	34.0 [3]	38.5 [2]	52.4 [3]	–	64.3 [1]	2.25
	LLL	62.8 [4]	65.6 [3]	76.1 [2]	75.2 [1]	–	2.5	32.0 [4]	44.7 [1]	61.5 [2]	63.1 [1]	–	2
APG	AIMed	–	66.0 [2]	**77.7** [1]	73.1 [2]	82.7 [2]	1.75	–	22.5 [4]	61.6 [4]	22.5 [4]	17.6 [4]	4
	**BioInfer**	71.2 [2]	–	76.0 [2]	**81.4** [1]	82.0 [3]	2	37.9 [1]	–	62.1 [3]	70.7 [1]	76.3 [1]	1.5
	HPRD50	72.3 [1]	58.2 [4]	–	71.2 [4]	56.9 [4]	3.25	34.2 [3]	40.7 [2]	–	60.9 [3]	62.1 [2]	2.5
	IEPA	70.1 [3]	**72.7** [1]	75.5 [3]	–	85.8 [1]	2	34.7 [2]	37.0 [3]	63.9 [1]	–	61.8 [3]	2.25
	LLL	61.1 [4]	65.7 [3]	73.5 [4]	72.9 [3]	–	3.5	32.0 [4]	45.8 [1]	63.0 [2]	63.0 [2]	–	2.25

In brackets we present the ranking of each training corpus for each kernel, and we also show the average rank of the training corpus over the 4 test corpora. Bold typeface indicates our best overall result for a corpus.
