# Supplementary material for: A Comprehensive Benchmark of Kernel Methods to Extract Protein–Protein Interactions from Literature
Source: PLoS Comput Biol. 2010 Jul 1;6(7):e1000837. doi: 10.1371/journal.pcbi.1000837 (PMC2895635; doi:10.1371/journal.pcbi.1000837)
Supplement: Table S7 — Cross-learning experiments with some selected kernel performed on 4 corpora (all but AIMed). Cross-learning experiments with some selected kernel performed on 4 corpora (all but AIMed). Classifiers are trained on the ensemble of three corpora and tested on the forth one. (0.07 MB PDF) [file pcbi.1000837.s007.pdf]

Table S7. Cross-learning experiments with some selected kernel performed on 4 corpora (all but AIMed)

Kernel	Test	AUC	ΔAUC	P	R	F	ΔF
SL	BioInfer	**73.6**	−1.3	54.5	46.3	50.1	3.9
	HPRD50	76.8	−1.2	53.1	79.1	63.5	1.3
	IEPA	77.0	1.4	64.5	67.2	65.8	5.4
	LLL	82.0	2.5	75.0	78.7	76.8	10.4
kBSPS	BioInfer	**73.6**	0.3	42.5	72.0	**53.5**	5.9
	HPRD50	78.7	0.4	59.6	85.9	**70.4**	3.0
	IEPA	82.1	1.1	67.5	80.6	**73.5**	2.8
	LLL	**86.5**	−0.3	76.7	88.4	**82.2**	3.1
APG	BioInfer	**72.7**	3.1	53.1	40.6	46.0	6.9
	HPRD50	**80.6**	−3.4	54.9	78.5	64.6	−5.1
	IEPA	**84.0**	1.6	76.3	64.5	69.9	10.3
	LLL	**86.1**	−0.4	77.7	82.9	80.2	7.9

Classifiers are trained on the ensemble of three corpora and tested on the forth one. Same parameters are used as in Table 3. Rows correspond to the test corpus. We indicated the difference in AUC and F-score compared to the 5 corpora CL setting (Table 3). Bold typeface shows our best results for a particular corpus (differences under 1 base point are ignored).
