# Supplementary material for: A Comprehensive Benchmark of Kernel Methods to Extract Protein–Protein Interactions from Literature
Source: PLoS Comput Biol. 2010 Jul 1;6(7):e1000837. doi: 10.1371/journal.pcbi.1000837 (PMC2895635; doi:10.1371/journal.pcbi.1000837)
Supplement: Table S8 — CV results with transductive SVM for kBSPS, edit, cosine kernels. Results with the transductive learning strategy for some selected kernels. (0.06 MB PDF) [file pcbi.1000837.s008.pdf]

Table S8. CV results with transductive SVM for kBSPS, edit, cosine kernels

Kernel	Corpus	AUC	P	R	F
kBSPS	AIMed	74.8 (−0.3)	44.0 (−6.1)	47.3 (+5.9)	45.0 (+0.4)
	BioInfer	76.6 (+1.4)	53.8 (+3.9)	60.6 (−1.2)	56.7 (+1.6)
	HPRD50	75.8 (−3.5)	62.9 (+0.7)	67.9 (−19.2)	64.0 (−7.0)
	IEPA	83.0 (−0.2)	63.9 (+5.1)	81.7 (−8.0)	71.2 (+0.7)
	LLL	81.1 (−3.2)	76.9 (+7.6)	84.8 (−8.4)	79.2 (+1.1)
cosine	AIMed	67.3 (−3.2)	39.0 (−4.6)	41.2 (−1.8)	39.6 (−1.3)
	BioInfer	64.2 (−1.9)	42.6 (−2.2)	45.2 (+1.2)	43.5 (−0.7)
	HPRD50	71.1 (−3.7)	60.3 (+1.3)	62.3 (−4.9)	60.5 (−0.7)
	IEPA	75.2 (−0.3)	63.8 (+2.5)	65.8 (−2.6)	64.1 (+0.0)
	LLL	75.5 (+0.3)	72.7 (+2.5)	66.2 (−15.5)	67.9 (−5.9)
edit	AIMed	73.4 (−1.8)	50.9 (−17.9)	49.8 (+22.1)	49.8 (+10.0)
	BioInfer	71.9 (+4.5)	51.0 (+0.6)	52.9 (+13.7)	51.3 (+7.5)
	HPRD50	71.0 (−8.2)	62.9 (−8.6)	61.4 (+16.2)	61.0 (+7.7)
	IEPA	75.9 (−4.3)	67.4 (−9.8)	68.7 (+8.5)	67.4 (−0.3)
	LLL	82.4 (−5.1)	81.3 (+13.7)	73.9 (−24.1)	75.1 (−3.3)

Average best results are shown. In parenthesis we show the difference compared to the results of standard SVM (Table 2).
