# Supplementary material for: A Comprehensive Benchmark of Kernel Methods to Extract Protein–Protein Interactions from Literature
Source: PLoS Comput Biol. 2010 Jul 1;6(7):e1000837. doi: 10.1371/journal.pcbi.1000837 (PMC2895635; doi:10.1371/journal.pcbi.1000837)
Supplement: Table S9 — Average runtime of training and test processes, and runtime estimates on entire Medline. Average runtime of training and test processes per corpus measured over all cross-validation experiments for each kernel (not including the parsing time at pre-processing), and rough runtime estimates on the entire Medline. (0.06 MB PDF) [file pcbi.1000837.s009.pdf]

Table S9. Average runtime of training and test processes per corpus measured over all cross-validation experiments for each kernel (not including the parsing time at pre-processing), and rough runtime estimates on the entire Medline

Kernel	Corpora (sec)										average test time per sentence	Medline (days)		
	AIMed		BioInfer		HPRD50		IEPA		LLL			estimated parsing time	estimated test time	estimated total time
	train	test	train	test	train	test	train	test	train	test				
SL	66.4	10.8	142.5	24.0	4.1	1.2	9.3	1.8	3.5	1.1	0.107	6	141	147
ST	142.0	18.0	459.9	60.3	0.5	0.1	2.2	0.3	0.9	∼0.0	0.130	1431	172	1603
SST	138.1	19.1	479.3	67.8	0.5	0.1	2.2	0.4	0.9	∼0.0	0.146	1431	192	1623
PT	1462.7	188.5	6088.7	832.6	8.5	1.3	39.7	7.0	13.5	1.0	1.779	1431	2342	3773
SpT	232.4	44.5	330.6	79.8	0.4	0.1	1.8	0.6	0.3	0.1	0.197	1431	259	1609
kBSPS	5.4	0.4	17.8	1.5	∼0.0	∼0.0	0.2	∼0.0	∼0.0	∼0.0	0.003	180	4	185
cosine	185.1	9.6	1853.6	31.7	1.3	0.1	4.0	0.3	0.9	∼0.0	0.070	180	92	272
edit	180.9	13.0	1073.1	27.7	0.8	0.1	3.0	0.5	0.7	0.1	0.070	180	92	272
APG	4517.4	3.7	8264.3	6.2	71.4	0.4	275.6	0.8	74.7	0.4	0.034	180	45	226
dict	223.9		465.1		11.4		33.6		16.6					
lin	203.1		404.8		10.6		30.7		13.9					
norm	50.7		87.1		3.6		8.1		3.6					
train	4039.7		7307.4		45.9		203.2		40.6					

Estimates on Medline are based on the followings: (1) Medline contains 10.35M articles with abstract and 7.65M with only title, on average 10.25 sentences per abstract; (2) parsing runtimes are estimated based on [38, Table 2] (syntax and dependency parsers) and [72, Table 2] (time ratio of shallow and various deep parsers). Numbers are given in seconds for benchmark corpora and days for Medline.
