# Supplementary material for: A Comprehensive Benchmark of Kernel Methods to Extract Protein–Protein Interactions from Literature
Source: PLoS Comput Biol. 2010 Jul 1;6(7):e1000837. doi: 10.1371/journal.pcbi.1000837 (PMC2895635; doi:10.1371/journal.pcbi.1000837)
Supplement: Text S1 — Similarity function in kBSPS kernel. Definition of similarity score used in kBSPS kernel. (0.07 MB PDF) [file pcbi.1000837.s010.pdf]

Similarity function in kBSPS kernel

The tolerant score function (t-score) is defined on two vertex-walks, t and s, of equal length q:

$$\text{t-score}_{L,E,D,l,e,d}(t,s) =$$
$$\begin{cases} 0, & \text{if } \big(L=-1 \text{ and } \exists i: t[i] \neq s[i] \text{ and type}(t[i],s[i]) = l\big) \\ & \text{ or } \big(E=-1 \text{ and } \exists i: t[i] \neq s[i] \text{ and type}(t[i],s[i]) = e\big) \\ & \text{ or } \big(E=-1 \text{ and } \exists i: \text{type}(t[i]) \text{ or type}(s[i]) \text{ is entity and type}(t[i],s[i]) = 0\big) \\ & \text{ or } \big(D=-1 \text{ and } \exists i: t[i] \neq s[i] \text{ and type}(t[i],s[i]) = d\big) \\ \sum_{i=1}^{2q-1} \text{type}(t[i],s[i]) \cdot \delta(t[i],s[i]), & \text{otherwise} \end{cases}$$

where $\text{type}(t[i]) = \{\text{surface,entity,dependency}\}$ is the type of $t[i]$, the ith element of the v-walk t,

$$\text{type}(t[i],s[j]) = \begin{cases} l, & \text{if type}(t[i]) = \text{type}(s[j]) = \text{surface} \\ e, & \text{if type}(t[i]) = \text{type}(s[j]) = \text{entity} \\ d, & \text{if type}(t[i]) = \text{type}(s[j]) = \text{dependency} \\ 0, & \text{otherwise} \end{cases}$$

assigns the token-type-dependent weight if the tokens are of the same type, and

$$\delta(t[i],s[j]) = \begin{cases} 1, & \text{if } t[i] = s[j] \\ 0, & \text{otherwise} \end{cases}$$

is the Kronecker-delta defined on tokens. Figure 3 shows some examples for the t-score calculation.
