# Supplementary material for: A Comprehensive Benchmark of Kernel Methods to Extract Protein–Protein Interactions from Literature
Source: PLoS Comput Biol. 2010 Jul 1;6(7):e1000837. doi: 10.1371/journal.pcbi.1000837 (PMC2895635; doi:10.1371/journal.pcbi.1000837)
Supplement: Text S2 — Additional experiments. We provide here details of two additional experiments. (1) Cross-learning (CL) without AIMed, that is systems are trained on 3 corpora and tested on the fourth one. (2) Models trained with transductive SVM. (0.05 MB PDF) [file pcbi.1000837.s011.pdf]

Additional experiments

Cross-learning without AIMed

We observed that the models trained on AIMed are the worst when applied to other corpora. Therefore, we investigated how CL performance (Table 3) of the best performing kernels changes when AIMed is discarded. The results in Table S7 show that AUC values change insignificantly, but F-scores increase by 3–6%. This experiment again proves that the difference between AIMed and the other 4 corpora is the largest, and that characteristics of a particular learning corpus have a considerable impact on performance.

Transductive SVM

Erkan et al applied the SVM with transductive learning strategy, which improved the F-score due to its higher recall especially for edit kernel [22]. Transductive learning also takes into account test examples when building a model. First, labels are assigned to test examples using the standard SVM. Then, the transductive SVM (TSVM) optimizes the class separating surface by switching the labels of two selected test data so that the overall objective function of the learner improves. TSVM was found to be superior to SVM for text classification [74], and transductive version is a built-in feature in SVM-Light.

We investigated the effect of using the TSVM with three selected kernels: kBSPS, cosine, and edit (application to AGP would be more complicated since its implementation is not based on SVM). The results in Table S8 show that in some particular cases, the F-score gain is remarkable. This happens when the difference between the original recall and precision values is large, and the F-score is dominated by the lower value. TSVM in those cases moves the separating surface towards the P/R break-even point which improves F-score. AUC is in general less sensitive to slight changes in the location of the separating surface, and, consequently, our observed AUC hardly changes (and if yes, then mostly negative). Overall, our results give no clear argument for the advantage of transductive learning, especially when an application needs either higher recall or higher precision. Note that TVSM drastically increases training time.
