# Supplementary material for: A Comprehensive Benchmark of Kernel Methods to Extract Protein–Protein Interactions from Literature
Source: PLoS Comput Biol. 2010 Jul 1;6(7):e1000837. doi: 10.1371/journal.pcbi.1000837 (PMC2895635; doi:10.1371/journal.pcbi.1000837)
Supplement: Text S3 — Theoretical complexity of kernels. We provide here details on the computational complexity of kernels. (0.04 MB PDF) [file pcbi.1000837.s012.pdf]

## Theoretical complexity of kernels

The amount of substructures extracted from a given representation differs substantially between the presented kernels. This has naturally a strong impact on their computational complexity. For the ST kernel, a linear complexity algorithm was proposed in [30]. Collins and Duffy have proven [20] that the SST kernel complexity is quadratic in the number of tree nodes. Moschitti [28] reports that the PT kernel also has a quadratic complexity, however, he claims that the running time is linear in the average case. It is proven [58] that the SpT kernel can be computed in linear time with respect to the number of tree nodes. Since, kBSPS kernel uses also v-walks as SpT, it has the same complexity (with the default $k = 0$, it is linear in the number of nodes in the shortest path). The cosine kernel is also linear, while the edit is quadratic in the number of nodes in the shortest path. APG uses the most complicated sentence structure encoding, which necessitates some expensive preprocessing steps. Only the calculation of the graph encoding containing matrix inversion is cubic in the number of nodes.
